# A Hybrid Search Behavior-Based Adaptive Grey Wolf Optimizer for Cooperative Path Planning for Multiple UAVs

**DOI:** 10.3390/s25247657

**Published:** 2025-12-17

**Authors:** Zhiwen Zheng, Hao Huang, Chenbo Li, Yongbin Yu, Xiangxiang Wang, Jingye Cai, Xi Huang, Songbo Hu

**Affiliations:** 1 School of Information and Software Engineering, University of Electronic Science and Technology of China, Chengdu 610054, China; 202212090803@std.uestc.edu.cn (Z.Z.); ybyu@uestc.edu.cn (Y.Y.); xxwang@uestc.edu.cn (X.W.); jycai@uestc.edu.cn (J.C.); 2 Electrical Engineering & Compute Sciences, University of California Berkeley, Berkeley, CA 94720, USA; hao_huang@berkeley.edu; 3 Chengdu Pvirtech Technology Co., Ltd., Chengdu 610000, China; huangxi@pvirtech.com (X.H.); husongbo@pvirtech.com (S.H.)

**Keywords:** swarm intelligence algorithm, cooperative path planning, multiple UAVs, hybrid search, grey wolf optimizer, multi-constraint problem

## Abstract

Cooperative path planning of multiple unmanned aerial vehicles (UAVs) is pivotal for improving mission efficiency and safety in complex scenarios. However, the multi-constraint of UAVs increases the design difficulity of cooperative path planning. To address these issues, a hybrid search behavior-based adaptive grey wolf optimizer (HSB-GWO) is proposed in this work. HSB-GWO incorporates three key innovations: (1) A dimension learning-based hunting (DLH) strategy is employed to enhance population diversity by enabling knowledge exchange between non-leader wolves and their neighbors. (2) Aquila exploration combining expand exploration for global potential region detection and Lévy flight-based narrowed exploration for preventing populations from falling into local optimal solutions is adopted to enrich search behaviors and avoid local optima. (3) An adaptive weight adjustment mechanism is designed for leader wolves (α, β, and δ) to dynamically tune their contribution to offspring generation based on fitness to improve high-quality solution utilization. The search performance of HSB-GWO on the benchmark functions was validated by experiments on the benchmark suites of IEEE CEC 2017 and 2019, in which HSB-GWO outperformed seven comparison algorithms (AO, AOA, CBOA, NOA, GWO, IGWO, and AGWO), with Friedman test confirming its top overall rank (Rank 1). The results of cooperative path planning simulation demonstrate that the high-quality multi-UAV trajectories can be generated by the HSB-GWO to guide UAVs from the start to the destination safely and smoothly with the smallest cost.

## 1. Introduction

The characteristics of high maneuverability, low operational cost, and the ability to perform tasks in complex environments mean that unmanned aerial vehicles (UAVs) are widely applied in both civil and military fields, such as disaster rescue, environmental monitoring, and cooperative reconnaissance [1,2,3]. Among various UAV-related technologies, cooperative path planning for multiple UAVs stands out as a core challenge, as it directly determines the efficiency, safety, and success rate of multi-UAV mission execution. Simultaneously designing the flight trajectories of multiple UAVs in parallel is a key challenge for this task. In addition to considering various environmental constraints (maximum and minimum flight) and threat avoidance (radar, missile, and artillery threats), it is also necessary to consider collisions between multiple UAVs and spatiotemporal coordination [4,5,6,7,8,9]. Therefore, the design of the path is complex.

The A* algorithm [10] and Dijkstra algorithm [11], as traditional and classical path planning methods, are limited by handling high-dimensional and complex constraint spaces, especially when dealing with dynamic threats and multi-UAV collaboration [12,13,14]. Due to the characteristics of strong search capabilities for the high-dimension solution space and flexibility in dealing with nonlinear constraints [15,16], metaheuristic algorithms have been widely adopted to emerge promising solutions for optimization of various application scenarios [17,18,19]. The grey wolf optimizer (GWO), proposed by Mirjalili et al., is a representative metaheuristic inspired by the social hierarchy and cooperative hunting behavior of grey wolves [20]. It has been widely used in UAV path planning due to its simple structure and few control parameters [21,22,23]. However, the standard GWO still has inherent shortcomings: its search performance is highly dependent on the three leader wolves (α, β, and δ), which easily leads to premature convergence and trapping in local optima when facing complex optimization tasks (e.g., multi-UAV cooperative path planning with multiple threats and time–space constraints) [24,25].

To address the limitations of GWO, numerous improved versions have been proposed [26,27,28]. To mitigate the insufficient exploration and premature convergence of the original GWO, Zhu et al. [26] adopted chaotic maps integrated into the position update process of GWO. Moreover, differential evolution (DE) combined with a fractal-based multi-scale search strategy is used to realize search with multiple levels of detail to enhance the exploitation ability of GWO. In [27], Liu et al. integrated Gaussian mutation and dynamic weights of trigonometric functions to adjust the importance of information for three leaders through differentiated weight adjustment. In addition, a spiral function was employed to perturb the optimal individual position, which prevented GWO from getting stuck in local optimal solutions. Due to the traditional GWO relying on leader wolves’ (α, β, and δ) guidance for population updates, the population tends to converge in the later stages of iteration, resulting in insufficient diversity and difficulty in breaking through local optima. Zhou [28] directly integrated crossover and mutation operators of the genetic algorithm (GA) into the GWO population update process, breaking population homogenization through gene recombination and random mutation, thereby improving premature convergence problems. Nevertheless, these improved algorithms still struggle to balance exploration and exploitation in complex solution spaces, such as hybrid and composite benchmark functions or multi-UAV path planning scenarios with dense threats. The imbalance between exploration and exploitation can lead to different inefficient search problems. Excessive exploration makes it difficult for individuals to achieve precise search within the existing search area, thereby impeding the approach of the global optimal solution. Conversely, excessive exploitation behavior causes inefficient searching for unexplored areas, resulting in falling into local optima, especially when the solution space contains multiple local optima [29,30]. In addition, existing path planning algorithms for cooperative path planning often ignore the coupling relationship between individual UAV trajectory optimization and global team coordination. For example, some algorithms focus on avoiding threats but may result in excessive flight distance and energy consumption, while others prioritize time coordination but cannot ensure collision avoidance between UAVs [31]. Therefore, there is an urgent need for a metaheuristic algorithm that can not only balance exploration and development to handle complex optimization tasks, but also effectively integrate multiple drone constraints (time coordination, spatial collision avoidance, threat avoidance) to generate high-quality cooperative trajectories.

Against this background, this study proposes a hybrid search behavior-based adaptive GWO (HSB-GWO) for multi-UAV cooperative path planning. The algorithm introduces dimension learning-based hunting (DLH) and Aquila exploration strategies to enrich the search behavior of the wolf pack, and adopts an adaptive weight adjustment mechanism for leader wolves to improve the utilization of high-quality solutions. By constructing a comprehensive cost model that integrates energy consumption, altitude constraints, threat avoidance, time coordination, and collision avoidance, HSB-GWO is expected to solve the multi-UAV cooperative path planning problem more effectively. The performance of HSB-GWO is verified through extensive experiments on IEEE CEC 2017 benchmark functions and a multi-UAV cooperative path planning scenario, providing a new efficient solution for multi-UAV path planning.

The remainder of this article is structured as follows. Section 2 introduces the modeling of cooperative path planning for multiple UAVs. Section 3 designs the proposed HSB-GWO. Section 4 reports the simulation results. The conclusion and future work are summarized in Section 5 and Section 6, respectively.

## 2. Modeling of Cooperative Path Planning for Multiple UAVs

In this part, the modeling of cooperative path planning task with various threats and constraints is introduced.

### 2.1. Terrain Modeling

Firstly, a three-dimensional geographic environment (planning space) is modeled by discretization. By dividing the planning space into cubic grids, the space can be reconstituted to numerous adjacent cubes of equal size. Based on the number of flight waypoints set, multiple waypoints are searched in the planning space in an orderly manner, connected from the starting point to the target point, forming a trajectory. The planning space is modeled by a two-dimensional matrix, where each element of the matrix represents the highest altitude of the corresponding cubic grids. Based on the above, the planning space *O* can be formed as

(1) $$\begin{array}{cc} O & =\left[\begin{array}{cccc} h_{1,1} & h_{1,2} & \cdots & h_{1,M_{O}}\\ h_{2,1} & h_{2,2} & \cdots & h_{2,M_{O}}\\ \vdots & \vdots & \ddots & \vdots \\ h_{N_{O},1} & h_{N_{O},2} & \cdots & h_{N_{O},M_{O}} \end{array}\right], \end{array}$$

in which $N_{O}$ and $M_{O}$ denote the size of the discretized planning space.

### 2.2. Constraint Design

#### 2.2.1. Threat Model Design

The threat model has constraints such as maximum effective range and effective kill distance. A reasonable target cost function is established to introduce these constraint conditions into the target function to form radar, missile, anti-aircraft gun, and atmospheric threat models. The model definitions for different threats in this paper are summarized below.

**Radar threat model**

We define $r_{\max}^{R}$ as the maximum scanning radius of the radar with the antenna performing a $360^{\circ }$ scan in azimuth, and the detection probability of radar for a UAV can be approximately expressed as

(2) $$T_{R}\left(D_{R}\right)=\left\{\begin{array}{cc} 0, & D_{\max}^{R}<D_{R}\\ \frac{1}{D_{R}^{4}}, & D_{\min}^{R}\leq D_{R}\leq D_{\max}^{R}\\ 1, & D_{R}<D_{\min}^{R} \end{array},\right.$$

in which $D_{R}$, $D_{\max}^{R}$, and $D_{\min}^{R}$ denote the distance between the UAV and the radar, the maximum radius of the radar detection area (the return signal will be too weak to recognize the UAV if $D_{\max}^{R}<D_{R}$), and the effective detection radius of the radar, respectively. $T_{R}\left(D_{R}\right)$ is defined as the probability of radar threat.

**Other threat models**

In addition to radar threats, there are also missile, artillery, and meteorological threats. Since the mathematical models of the three threats mentioned above are the same, they are uniformly defined as other threats. The probability of other threats can be formed as

(3) $$T_{S}\left(D_{j}\right)=\left\{\begin{array}{cc} 0, & D_{\max}^{S}<D_{j}\\ \frac{1}{D_{j}}, & D_{\min}^{S}\leq D_{j}\leq D_{\max}^{S}\\ 1, & D_{j}<D_{\min}^{S} \end{array},\right.$$

in which $D_{j}\in D_{S}$ ($D_{S}=\left\{D_{M},D_{A},D_{C}\right\}$) denotes the distance belonging to missile, artillery fire, and meteorological threats.

#### 2.2.2. Collaborative Constraints

In addition to the constraints for a single UAV mentioned above, further consideration is needed for the constraints among UAVs to ensure the entire UAV formation can successfully complete the task. Therefore, two types of collaborative constraints will be introduced in the following.

**Time collaborative constraint**

For the *m*-th UAV, assuming that its trajectory length is $L_{m}$ (m=1,2,⋯,M), its corresponding time constraint can be designed through the maximum and minimum arrival time as

(4) $$\begin{array}{c} t_{\min}^{m}\leq t_{m}\leq t_{\max}^{m} \end{array}$$

where $t_{\min}^{m}$ and $t_{\max}^{m}$ are the shortest and longest arrival time for the *m*-th UAV calculated by the corresponding maximum and minimum velocities as

(5) $$\left\{\begin{array}{c} t_{\min}^{m}=\frac{L_{m}}{v_{\max}^{m}}\\ t_{\max}^{m}=\frac{L_{m}}{v_{\min}^{m}} \end{array},\right.$$

**Space collaborative constraint**

Space collaborative constraint, also known as collision-free constraint, requires that the minimum distance between UAVs is not less than the minimum safe flight distance. For the *m*-th UAV, the distance to the *n*-th UAV need to be satisfied with

(6) $$\begin{array}{c} D_{m,n}>D_{K},\;n=1,2,\cdots ,m-1 \end{array}$$

in which $D_{m,n}$ is the distance between the two UAVs and $D_{K}$ is defined as the minimum safe flight distance among the UAVs.

### 2.3. Cost Model for Trajectory Planning of UAVs

In the collaborative flight mission of multiple UAVs, trajectory evaluation needs to further introduce indicators relating the time and space coordination on the basis of the single-UAV cost function. Thus, the cost function for UAVs can be formed as

(7) $$\begin{array}{cc} F=\sum_{m=1}^{M}(\omega_{1}f_{E,m}+ & \omega_{2}f_{H,m}+\omega_{3}f_{T,m}+\omega_{4}f_{F,m}+\omega_{5}f_{C,m}) \end{array}$$

in which $\left\{\omega_{1},\cdots ,\omega_{5}\right\}$ are weight factors, $f_{E,m},$$f_{H,m},$$f_{T,m},$$f_{F,m},$ and $f_{C,m}$ are the energy consumption, altitude, threats, flight time, and collision cost for the *m*-th UAV, respectively.

For the energy consumption, $f_{E,m}$ can be computed by

(8) $$\begin{array}{c} f_{E,m}=p_{E}\sum_{d=1}^{D_{m}-1}l_{d,m}, \end{array}$$

where $p_{E}$ is defined as a proportional factor (the fuel consumption per kilometer) and $l_{d}$ denotes the *d*-th distance of flight path formed as

(9) $$\begin{array}{cc} l_{d,m}=[{\left(x_{d+1,m}-x_{d,m}\right)}^{2} & +{\left(y_{d+1,m}-y_{d,m}\right)}^{2}+{\left(z_{d+1,m}-z_{d,m}\right)}^{2}{]}^{1/2} \end{array}$$

in which *d* is defined as the *d*-th waypoint; $\left(x_{d,m},y_{d,m},z_{d,m}\right)$ is the spatial coordinate of the *m*-th UAV at the *d*-th waypoint.

For the altitude, $f_{H,m}$ is formed as

(10) $$\begin{array}{c} f_{H,m}=\sum_{d=1}^{D_{m}}u_{d}, \end{array}$$

in which $u_{d}$ is the punish function for the *d*-th waypoint of *m*-th UAV that can be described as

(11) $$u_{d}=\left\{\begin{array}{cc} p_{H_{1}}\left(z_{d,m}-H_{\max}\right), & \;\mathrm{if}\;H_{\max}<z_{d,m}\\ 0, & \;\mathrm{if}\;H_{\min}\leq z_{d,m}\leq H_{\max}\\ p_{H_{2}}\left(H_{\min}-z_{d,m}\right), & \;\mathrm{if}\;0\leq z_{d,m}<H_{\min} \end{array},\right.$$

in which $H_{\min}$ and $H_{\max}$ are the minimum and maximum altitude for UAV flight. $p_{H_{1}}$ and $p_{H_{2}}$ are proportional factors.

$f_{T,m}$ as a threat cost can be computed by

(12) $$\begin{array}{c} f_{T,m}=\sum_{d=1}^{D_{m}}T_{R}\left(D_{R,d,m}\right)+\sum_{j=1}^{3}\left(\sum_{d=1}^{D_{m}}T_{S}\left(D_{j,d,m}\right)\right), \end{array}$$

where $D_{i,d,m}\in \left\{D_{R,d,m},D_{S,d,m}\right\}$ can be calculated by

(13) $$\begin{array}{cc} D_{i,d,m}=[{\left(x_{d,m}-x_{i}\right)}^{2} & +{\left(y_{d,m}-y_{i}\right)}^{2}+{\left(z_{d,m}-z_{i}\right)}^{2}{]}^{1/2} \end{array}$$

in which $\left(x_{i},y_{i},z_{i}\right)$ is defined as the center position coordinate of radar and other threats.

According to the information on the time collaborative constraint, time cost $f_{F,m}$ can be modeled as

(14) $$f_{F,m}=\left\{\begin{array}{cc} 0, & \;\mathrm{if}\;t_{\min}^{m}\leq t_{\mathrm{com}}\leq t_{\max}^{m}\\ \left|t_{m}-t_{\mathrm{com}}\right|, & \;\mathrm{otherwise} \end{array},\right.$$

in which $t_{m}$ is the actual arrival time of *m*-th UAV and $t_{\mathrm{com}}$ is the instruction time. When the range of theoretical flight time $\left[t_{\min}^{m},t_{\max}^{m}\right]$ for *m*-th UAV does not include $t_{\mathrm{com}}$, it indicates that the *m*-th UAV is too fast or too slow to meet the time collaborative requirement.

Each UAV will fly at a constant speed $v_{m}$, and the flight time $t_{m}$ can be calculated with the trajectory determined by waypoints (Equation (9)), which can be formed as

(15) $$\begin{array}{c} t_{m}=\frac{1}{v_{m}}\sum_{d=1}^{D_{m}-1}l_{d,m}. \end{array}$$

The collision cost $f_{C,m}$ can be calculated based on the distance between the *m*-th UAV and the 1st to (m−1)-th UAVs throughout the entire flight; collision can be considered to occur if Equation (6) is not satisfied.

**Remark** **1.** *Considering that threat areas with spherical shapes are common in real environments, the aforementioned threat models are constructed. It is worth noting that threat modeling and HSB-GWO are independent, which means that different modeling methods will not affect the path planning algorithm. Therefore, different models and penalty functions can be designed according to actual needs.*

**Remark** **2.** *In addition to adding weight factors to convert multiple indicators into a single fitness function (Equation (7)), constructing a multi-objective architecture is another strategy to design the optimization task. Under the multi-objective architecture, the metaheuristic algorithm is conducted to search the Pareto frontier composed of multiple indicators, and there is no dominant relationship between the solutions on this frontier, which can be considered as optimal solutions.*

### 2.4. Optimization Task Mathematical Formulation

Based on the previous content, the mathematical formulation of the optimization problem for the collaborative path planning can be summarized as follows:

(16) $$\begin{array}{c} \begin{array}{cc} \min_{X}F\left(X\right) & =\sum_{m=1}^{M}\left(\omega_{1}f_{E,m}+\omega_{2}f_{H,m}+\omega_{3}f_{T,m}+\omega_{4}f_{F,m}+\omega_{5}f_{C,m}\right)\\ \; & s.t.\;\left\{\begin{array}{c} H_{\min}\leq z_{d,m}\leq H_{\max},m\in \left[1,M\right],d\in \left[1,D_{m}\right]\\ v_{\min}\leq v_{m}\leq v_{\max},m\in \left[1,M\right]\\ D_{m,n}>D_{K},m\neq n,\left\{m,n\right\}\in \left[1,M\right]\\ t_{\min}^{m}\leq t_{\mathrm{com}}\leq t_{\max}^{m},m\in \left[1,M\right]\\ 0\leq x_{m,d}\leq M_{O}\\ 0\leq y_{m,d}\leq N_{O}\\ z_{m,d}\geq h_{n,m},h_{n,m}\in O \end{array}\right. \end{array} \end{array}$$

## 3. Hybrid Search Behavior-Based Adaptive Grey Wolf Optimizer

GWO, introduced by Mirjalili et al. [20], is inspired by the social hierarchy and cooperative hunting behavior of grey wolves. GWO emulates the leadership structure within a wolf pack, typically categorized into four levels, alpha (α), beta (β), delta (δ), and omega (ω), in which the α, β, and δ wolves, representing the fittest solutions, are regarded as leaders to guide the search process of the ω wolves that follow them. In [32], it is suggested that GWO is a variant algorithm of PSO (similar to SPSO-2011). Therefore, the shortcomings of GWO in search performance can reflect the common problems of PSO-based metaheuristic algorithms, which is the focus of this work to solve.

GWO mathematically models the strategies of encircling, hunting, and attacking prey. The positions of the search agents (wolves) are updated based on the perceived locations of the α, β, and δ wolves, simulating the collaborative effort of the pack to approach the optimal solution (prey). This mechanism is developed to balance the exploration of the search space and the exploitation of promising regions. However, the search performance of the whole population is strongly dependent on the three best-positioned wolves, which easily causes the population to fall into the local optimal solution and weak search stability.

**Figure 1:**
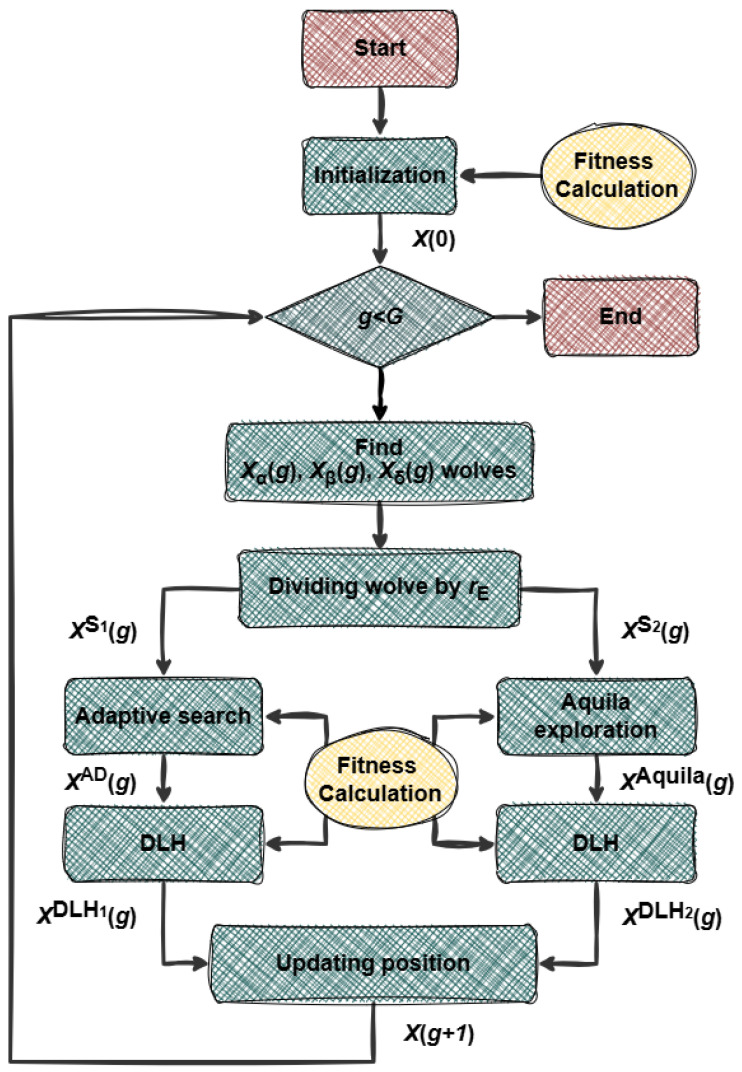
Calculation flow for the HSB-GWO.

In order to overcome the weakness of traditional GWO, in this paper a novel algorithm named hybrid search behavior-based adaptive GWO (HSB-GWO) is developed. Dimension learning-based learning (DLH) [33] and Lévy flight [34] are introduced into the GWO for improve the balance of exploration and exploitation behaviors. In addition, the adaptive strategy is also adopted to dynamically adjust the weights of the α, β, and δ wolves in offspring generation. The overall diagram of the HSB-GWO algorithm is depicted in Figure 1 and the pseudo-code is shown in Algorithm 1.
**Algorithm 1:** HSB-GWO
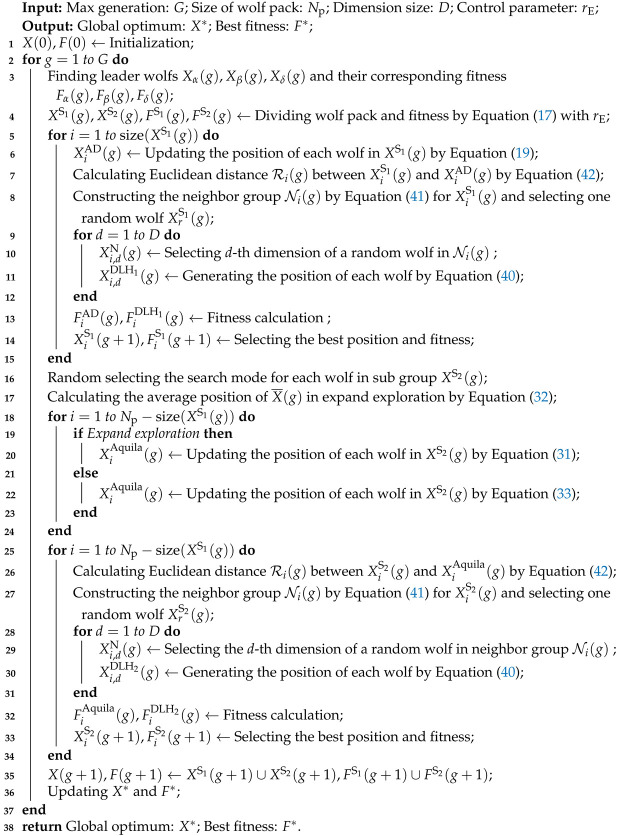

**Remark** **3.** *It is worth noting that HSB-GWO performs fitness calculation twice per generation (see lines 13 and 32 of Algorithm 1). Therefore, G of HSB-GWO should be set half compared to the other algorithms to ensure the fitness evaluations (FEs) are equal for fair comparison.*

### 3.1. Individual Formulation

**Figure 2:**
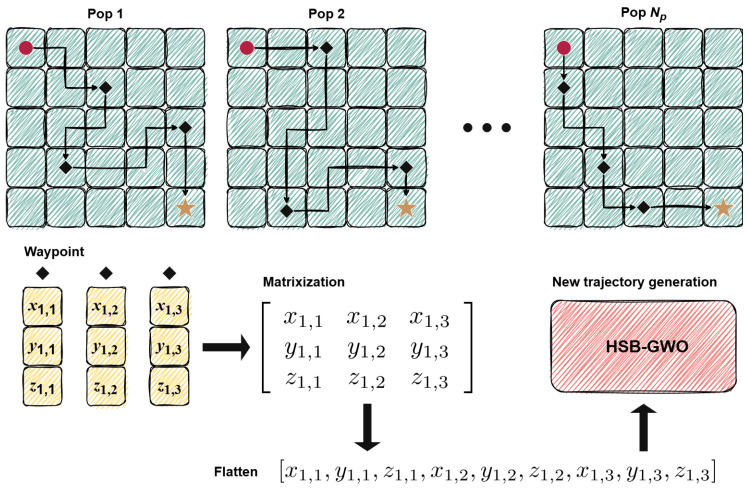
Example for the trajectory generation.

The cooperative path planning task for multiple UAVs is achieved by generating a certain number of waypoints on a three-dimensional map for each UAV. Therefore, each individual of HSB-GWO contains the coordinates of these waypoints. In addition, the search range of HSB-GWO for paths is determined by the size of the 3D map constructed (flight altitude range $\left[H_{\min},H_{\max}\right]$ and map area $N_{O}\times M_{O}$). Figure 2 is an example of path planning in a two-dimensional map.

**Figure 3:**
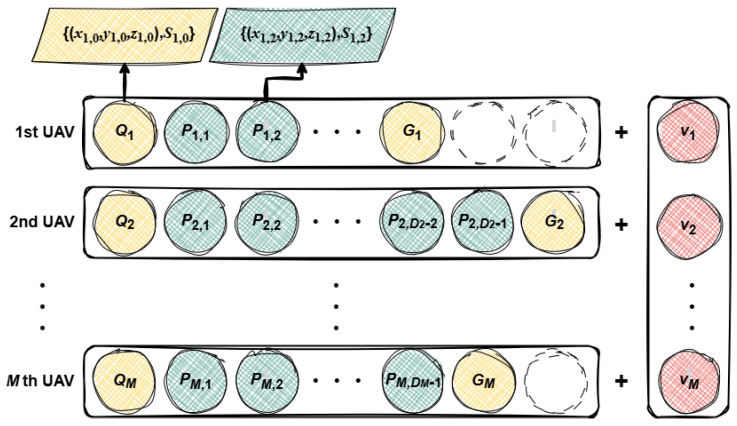
Collaborative track chromosome encoding.

The data structure of each wolf for multi-UAV path planning is illustrated in Figure 3, where a sequence of waypoints starting from the initial node $Q_{m}\;\left(m=1,2,\cdots ,M\right)$ and terminating at the goal waypoint $G_{m}$ is adopted to record spatial position information about each UAV for the calculation of $f_{E,m}$ and $f_{H,m}$. In addition, between $Q_{m}$ and $P_{m,D_{m}}$$\left(D_{m}=D_{1},D_{2},\cdots ,D_{M}-1\right)$, state variables $S_{m,0}$ (for $Q_{m}$) and $S_{m,D_{m}}$ (for $P_{m,D_{m}}$) are integrated into the information set to record constraint states (the number of collisions generated by the path formed by waypoints $\left\{Q_{m},P_{m,1}\right\}$ or $\left\{P_{m,D_{m}},P_{m,D_{m}+1}\right\}$, and the threat area passed through) for the calculation of $f_{T,m}$ and $f_{C,m}$. Each UAV has an independently set fixed speed. Therefore, the speed $v_{m}$ will also be recorded for the calculation of $f_{F,m}$.

According to the above content, HSB-GWO will generate waypoints and velocities for each UAV based on search strategies, then update the constraint state according to the coordinates of the waypoints and velocity, and finally record the relevant information in each individual for the calculation of the cost function, thereby quantifying the performance of the UAV under the cooperative path planning task.

### 3.2. Wolf Pack Dividing

Before the search of each iteration, wolves are divided into two sub groups $X^{S_{1}}\left(g\right)$ and $X^{S_{2}}\left(g\right)$ randomly by

(17) $$\left\{\begin{array}{cc} X_{i}\left(g\right)\in X^{S_{1}}\left(g\right),\; & \mathrm{if}\;\mathrm{rand}>r_{E}\\ X_{i}\left(g\right)\in X^{S_{2}}\left(g\right), & \mathrm{else} \end{array},\right.$$

in which $r_{E}$ is defined as the dividing probability, *i* denotes the *i*-th individual in the wolf pack ($i\leq N_{p}$), *g* is defined as the *g*-th iteration (g≤G), and rand is a random function to generate disturbance within [0,1].

### 3.3. Adaptive Search

Firstly, the surround behavior is conducted to update the position of the wolf pack that can be modeled by

(18) $$\begin{array}{cc} \; & D=|C\circ X_{P}\left(g\right)-X\left(g\right)| \end{array}$$

(19) $$\begin{array}{cc} \; & X^{\mathrm{AD}}\left(g\right)=X_{P}\left(g\right)-A\circ D \end{array}$$

where ∘ denotes the Hadamard product, $X_{P}\left(g\right)$ is defined as the position of the prey, X(g) denotes the position of the wolf in the *g*-th generation, *A* and *C* are the random coefficients, in which *A* is a control parameter to adjust the trend of the individual position movement and *C* is adopted to generate various distances from the population to the α, β, and δ wolves. The update laws of these two correlation coefficient vectors are designed as

(20) $$\begin{array}{cc} \; & A=2a\cdot r_{1}-a \end{array}$$

(21) $$\begin{array}{cc} \; & C=2\cdot r_{2} \end{array}$$

where $r_{1}$ and $r_{2}$ are random vectors generated within [0,1], and *a* is defined as the attenuation coefficient to decrease from 2 to 0 with increasing iterations.

**Figure 4:**
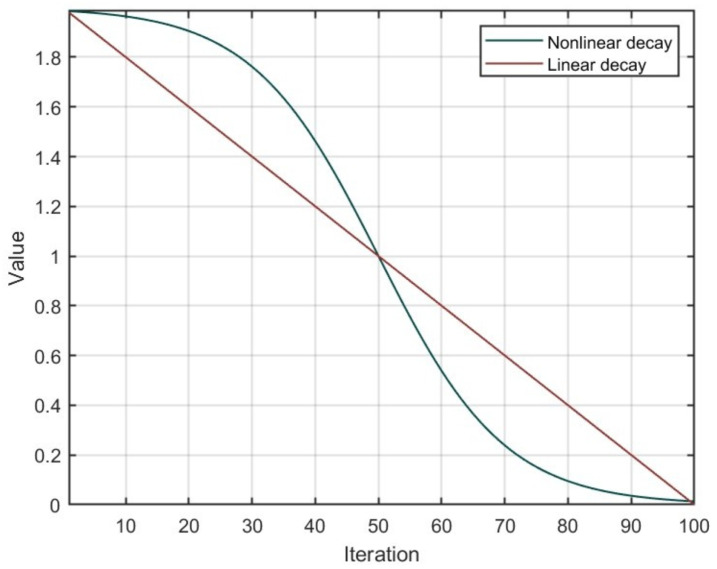
Convergence of nonlinear decay (Equation (22)) and linear decay.

Different from the linear decay adopted in the conventional GWO, the update law of *a* proposed in [35] is formed as

(22) $$\begin{array}{c} a=1+\tanh\left(2.5\left(1-\frac{2g}{G}\right)\right) \end{array}$$

in which *G* denotes the maximum iteration for the HSB-GWO. Figure 4 shows the convergence process of the linear decay method and Equation (22). Nonlinear decay can enrich the behavior of wolf packs throughout the entire search, enabling them to exhibit certain exploitation behavior in the early search stages to accelerate the convergence speed of the population. Similarly, in the later stages of the search, certain exploration behavior can also be exhibited, thereby reducing the possibility of falling into local optima.

The hunting stage is conducted after the positions of the leader wolves (α, β, and δ wolves) are updated. In wolf pack hunting, the leader wolves are the closest to the prey. Therefore, considering that α, β, and δ wolves have a better knowledge of the location of the optimal global solution, the other wolves are guided by them. The hunting behavior can be mathematically modeled by

(23) $$\begin{array}{cc} \; & D_{\alpha }=\left|C_{1}\circ X_{\alpha }-X\left(g\right)\right| \end{array}$$

(24) $$\begin{array}{cc} \; & D_{\beta }=\left|C_{1}\circ X_{\beta }-X\left(g\right)\right| \end{array}$$

(25) $$\begin{array}{cc} \; & D_{\delta }=\left|C_{1}\circ X_{\delta }-X\left(g\right)\right| \end{array}$$

where $C_{1}$, $C_{2}$, and $C_{3}$ are computed by Equation (21). |·| is a sign for absolute value. We substitute $D_{\alpha }$, $D_{\beta }$, and $D_{\delta }$ into Equation (18), which can be formed as

(26) $$\begin{array}{cc} \; & X_{i_{1}}\left(g\right)=X_{\alpha }\left(g\right)-A_{i_{1}}\circ D_{\alpha }\left(g\right) \end{array}$$

(27) $$\begin{array}{cc} \; & X_{i_{2}}\left(g\right)=X_{\beta }\left(g\right)-A_{i_{2}}\circ D_{\beta }\left(g\right) \end{array}$$

(28) $$\begin{array}{cc} \; & X_{i_{3}}\left(g\right)=X_{\delta }\left(g\right)-A_{i_{3}}\circ D_{\delta }\left(g\right) \end{array}$$

in which $X_{\alpha }\left(g\right)$, $X_{\beta }\left(g\right)$, and $X_{\delta }\left(g\right)$ are the top three solutions at the *g*-th generation; $A_{i_{1}}$, $A_{i_{2}}$, and $A_{i_{3}}$ are calculated by Equation (20).

In the conventional GWO, the generation of a new solution $X^{\mathrm{AD}}\left(g\right)$ is achieved by calculating the mean of $X_{i_{1}}\left(g\right)$, $X_{i_{2}}\left(g\right)$, and $X_{i_{3}}\left(g\right)$, which can be written as

(29) $$\begin{array}{c} X^{\mathrm{AD}}\left(g\right)=\frac{1}{3}\left(X_{i_{1}}\left(g\right)+X_{i_{2}}\left(g\right)+X_{i_{3}}\left(g\right)\right). \end{array}$$

However, by considering the differences in their fitness, their contribution to the generation of a new solution should also be different. For the current best solution $X_{\alpha }\left(g\right)$, the importance of its $X_{i_{1}}\left(g\right)$ should be the highest. Therefore, Equation (29) is rewritten as follows:

(30) $$\begin{array}{c} X^{\mathrm{AD}}\left(g\right)=\left(F_{i_{1}}\left(g\right)X_{i_{1}}\left(g\right)+F_{i_{2}}\left(g\right)X_{i_{2}}\left(g\right)+F_{i_{3}}\left(g\right)X_{i_{3}}\left(g\right)\right){\left(F_{i_{1}}\left(g\right)+F_{i_{2}}\left(g\right)+F_{i_{3}}\left(g\right)\right)}^{-1}, \end{array}$$

where $F_{i_{1}}\left(g\right)$, $F_{i_{2}}\left(g\right)$, and $F_{i_{3}}\left(g\right)$ are the fitness of $X_{\alpha }\left(g\right)$, $X_{\beta }\left(g\right)$, and $X_{\delta }\left(g\right)$. *F* represents the total cost generated during the flight process, in which $f_{E,m}>0$ always holds and the minimum values of other elements ($f_{H,m}$, $f_{T,m}$, $f_{F,m}$, and $f_{C,m}$) are 0. Therefore, F>0 always holds.

### 3.4. Aquila Exploration

Aquila exploration as a hybrid search method is proposed by Ma et al. [24], in which two search strategies are introduced to prevent wolves from falling into a local optimal solution. Moreover, hybrid search modes combined with the aforementioned adaptive search method can further enrich the diversity of wolves to improve search ability and stability.

#### 3.4.1. Expand Exploration

This search strategy is used to act as an investigator for locating the potential areas in the solution space, which can be formed as

(31) $$\begin{array}{c} X^{\mathrm{Aquila}}\left(g\right)=X_{\alpha }\left(g\right)\cdot \left(1-g/N_{g}\right)+\left(\bar{X}\left(g\right)-X_{\alpha }\left(g\right)\circ \mathrm{rand}\right), \end{array}$$

where $X^{\mathrm{Aquila}}\left(g\right)$ is defined as a new position for the wolf, $1-g/N_{g}$ is adopted as the linear decay factor to control search behavior and $\bar{X}\left(g\right)$ is the average value of all the positions of the wolves participating in the expand exploration that can be calculated by

(32) $$\begin{array}{c} \bar{X}\left(g\right)=\frac{1}{N_{E}}\sum_{i=1}^{N_{E}}X_{i}\left(g\right), \end{array}$$

in which $N_{E}$ denotes the number of wolves in expand exploration.

#### 3.4.2. Narrowed Exploration

This search mode imitates the hunting behavior of Aquila after discovering prey, wolves are expected to be able to perform local search behavior while possessing the ability to escape from local optima. Therefore, Levy flight with variable step size conforming to the characteristics of heavy tailed distribution is introduced. The new position updated by this search mode can be formed as follows:

(33) $$\begin{array}{c} X^{\mathrm{Aquila}}\left(g\right)=X_{\alpha }\left(g\right)\circ \mathrm{Levy}\left(D\right), \end{array}$$

where Levy(D) is calculated by

(34) $$\begin{array}{c} \mathrm{Levy}(D)=cS(\nu ), \end{array}$$

in which *c* represents a fixed constant (0.01) and the mathematical formula of S(ν) is written as

(35) $$\begin{array}{c} S\left(\nu \right)=\frac{1}{\pi }\int_{0}^{\infty }e^{-\varepsilon q^{\phi }}\cos\left(q\nu \right)dq,0.3\leq \phi \leq 1.99, \end{array}$$

which can be approximated and computed by the Mantegna method [36] as

(36) $$\begin{array}{cc} \; & S\left(\nu \right)=\frac{u}{{\left|v\right|}^{1/\phi }}, \end{array}$$

(37) $$\begin{array}{cc} \; & u∼N\left(0,\sigma_{u}^{2}\right),v∼N\left(0,\sigma_{v}^{2}\right), \end{array}$$

in which ϕ is a fixed constant set to be 1.5 and *u* is generated by a random function that conforms to a normal distribution with variance $\sigma_{u}^{2}$ computed by

(38) $$\begin{array}{c} \sigma_{u}={\left(\frac{\Gamma (1+\phi )\sin(\pi \phi /2)}{\Gamma \left(\frac{1+\phi }{2}\right)\phi \cdot 2^{(\phi -1)/2}}\right)}^{1/\phi }. \end{array}$$

Similarly, the variance of a random function for *v* is 1. Γ(ϕ) is a gamma function, which can be computed according to Weierstrass’s definition as

(39) $$\begin{array}{cc} \; & \Gamma \left(\phi \right)=\frac{e^{-\gamma \phi }}{\phi }\prod_{n=1}^{N}{\left(1+\frac{\phi }{n}\right)}^{-1}e^{\phi /n}\\ \; & s.t.\;{\left(1+\frac{\phi }{n}\right)}^{-1}e^{\phi /n}<10^{-12} \end{array}$$

where γ is the Euler–Mascheroni constant, 0.577216.

### 3.5. Dimension Learning-Based Hunting (DLH)

The unstable convergence caused by the generation of new individuals relying on the three leader wolves cannot be unavoidable in the conventional GWO. Moreover, hunting, as another interesting social behavior of the non-leader members, is neglected, which is potential information for enhancing search performance. Therefore, DLH is adopted to overcome these issues, which is an effective strategy to achieve knowledge learning for each wolf from its neighbors.

For the new position of each wolf, its *d*-th dimension $X_{i,d}^{\mathrm{DLH}}\left(g\right)$ can be calculated by

(40) $$\begin{array}{c} X_{i,d}^{\mathrm{DLH}}\left(g\right)=X_{i,d}\left(g\right)+\mathrm{rand}\cdot \left(X_{i,d}^{N}\left(g\right)-X_{r,d}\left(g\right)\right), \end{array}$$

in which $X_{i,d}\left(g\right)$ is the position of the *i*-th wolf, $X_{r,d}\left(g\right)\in X_{r}\left(g\right)$ is a wolf selected randomly from the current population X(g), and $X_{i,d}^{N}\left(g\right)$ is defined as the *d*-th dimension of a composite individual $X_{i}^{N}\left(g\right)$ combined by neighbor group $N_{i}\left(g\right)$. The neighbors of each wolf $X_{i}\left(g\right)$ represented by $N_{i}\left(g\right)$ are constructed as follows:

(41) $$\begin{array}{c} N_{i}\left(g\right)=\left\{X_{j}\left(g\right)|D_{i}\left(X_{i}\left(g\right),X_{j}\left(g\right)\right)\leq R_{i}\left(g\right),X_{j}\left(g\right)\in X\left(g\right)\right\}, \end{array}$$

in which $D_{i}\left(X_{i}\left(g\right),X_{j}\left(g\right)\right)$ is the Euclidean distance between $X_{i}\left(g\right)$ and $X_{j}\left(g\right)$. Similarly, $R_{i}\left(g\right)$, as the Euclidean distance, can be computed by

(42) $$\begin{array}{c} R_{i}\left(g\right)=∥X_{i}\left(g\right)-X_{i}^{\prime }\left(g\right)∥ \end{array}$$

where $X_{i}\left(g\right)$ is the current position of the *i*-th wolf and $X_{i}^{\prime }\left(g\right)$ denotes the new position of a wolf updated by the aforementioned two search strategies designed in Section 3.3 and Section 3.4.

## 4. Simulations

In this section, the searching performance of our proposed HSB-GWO algorithm is evaluated through several test functions and the simulation of UAVs for cooperative path planning. All the simulations were conducted on a CPU, Intel Core (TM) i9-13900HX 2.2 GHz and 64.00 GB RAM (Intel, Santa Clara, CA, USA), and the version of Matlab is R2023b.

### 4.1. Control Parameter Analysis

The search performance evaluations of the proposed HSB-GWO with different control parameter settings were performed by *IEEE Congress on Evolutionary Computation 2017 (IEEE CEC 2017)* benchmark suite consisting of 29 test functions [37]. These test suite combines unimodal (F1, F3), multimodal (F4–F10), hybrid (F11–F20), and composition (F21–F30) functions. All test functions were adopted with three dimensions of D=10, D=30, and D=50 by 10 independent runs. The number of fitness evaluations (FEs) was set based on $\left(D\times 10^{4}\right)$ for fair comparison. The value of population size is $N_{p}=100$.

The Friedman test [38] was used for ranking HSB-GWO with seven parameter settings based on their obtained fitness. As shown in Table 1, for the F1 and F3 group (unimodal functions), the mean ranks of HSB-GWO with $r_{E}=0.2$ are 1.00 (D=10), 2.00 (D=30), and 1.50 (D=50), which are relatively low in comparison to other $r_{E}$ settings. In the F4–F10 group (multimodal functions), the mean ranks for $r_{E}=0.2$ are 1.57 (D=10), 1.43 (D=30), and 2.29 (D=50), exhibiting superior performance over most other $r_{E}$ settings. Regarding the F11–F20 group (hybrid functions), the mean ranks with $r_{E}=0.2$ are 3.20 (D=10), 3.30 (D=30), and 2.40 (D=50), demonstrating competitiveness. In the F21–F30 group (composition functions), the mean ranks for $r_{E}=0.2$ are 3.30 (D=10), 2.20 (D=30), and 3.20 (D=50), indicating favorable results.

From the perspective of overall mean ranks across all function groups and dimensions, the HSB-GWO with $r_{E}=0.2$ obtained the lowest mean ranks (2.69 for D=10, 2.38 for D=30, and 2.59 for D=50) in contrast to other $r_{E}$ configurations. A lower mean rank signifies better algorithm performance. Moreover, considering the complexity of solution space for the cooperative path planning of multiple UAVs, $r_{E}=0.2$, which performs well in different types of benchmark functions, is chosen for subsequent experiments. The variation of control parameters can determine the search behavior of HSB-GWO; therefore, the performance of the algorithm in different search tasks can be improved by adjusting the control parameters.

**Remark** **4.** *In addition to finding the optimal configuration of control parameters through the above experiments, a variety of tuners, such as CRS-Tuning [39], F-Race [40], REVAC [41], and ParamILS [42], can also be adopted.*

### 4.2. Search Performance Comparison Experiment on Benchmark Functions

To validate the search performance of HSB-GWO, seven metaheuristic algorithms are adopted: AO [43], AOA [44], CBOA [45], NOA [46], GWO [20], IGWO [25], and AGWO [24]. All the control parameters of seven comparative algorithms were set following the recommended settings provided from their original works. Due to the varying number of fitness evaluation operators used by different algorithms in each iteration, the maximum number of fitness evaluations (MaxFEs) was set based on $\left(D\times 10^{4}\right)$. The value of population size is $N_{p}=100$. Two benchmark function sets (IEEE CEC 2017 and 2019) were adopted to verify the search performance of our proposed HSB-GWO.

The mean fitness errors (MFEs) representing the difference between the best fitness and the global optimum are summarized in Table 2, Table 3, Table 4, Table 5, Table 6 and Table 7. Moreover, standard deviations (STD) of fitness errors were also reported to measure the search performance of each algorithm. “w/t/l” denotes the number of wins (w), ties (t), and losses (l).

#### 4.2.1. Results of IEEE CEC 2017

**Exploitation and exploration ability analysis**

F1 and F3 as unimodal test functions are well-suited for verifying the exploitation capability to locate the optimal solution. The results shown in Table 2 demonstrate that the proposed HSB-GWO algorithm can yield highly competitive results on unimodal test functions. Notably, it significantly improved results on F3 across all dimensions (D=10,30,50) compared to the comparative algorithms. Hence, the effective exploitation ability of the HSB-GWO algorithm on the region around the optimal solution is proved.

Multimodal functions (F4–F10), which possess numerous local minima, can be utilized to test both the exploration ability and the local optimum avoidance capability of the HSB-GWO. According to the results presented in Table 3, HSB-GWO is capable of providing superior results on multimodal functions for different dimensions (D=10,30,50) in F4–F7 and F9, which indicates that the proposed HSB-GWO algorithm is competitive in terms of exploration.

**Search performance analysis on complex optimization tasks**

Optimization for the hybrid (F11–F20) and composite (F21–F30) functions possessing complex solution space requires strong and stable search performance of algorithms. Thus, the balance between exploitation and exploration of HSB-GWO can be evaluated simultaneously by these test functions.

The results summarized in Table 4 indicate that the HSB-GWO performs superiorly on all hybrid functions across three different dimensions (D=10, 30, 50) except F13 and F16. Although IGWO, as a comparative algorithm, achieved better results in F13 with D=10 and F16 with D=30, 50, the overall search performance of HSB-GWO is superior. Furthermore, Table 5 presents the solutions obtained by the HSB-GWO and other algorithms for solving composition functions (F21-F30). HSB-GWO won or tied all the other seven algorithms on F22-F24, F26, F29, and F30 with D=10, 30, 50. Although HSB-GWO lost to AO in F21 with D=10 and IGWO in F25 with D=10, F27 with D=30, and F28 with D=10, 50, its MFEs in these functions are close to the best. Table 6 summarizes the overall effectiveness of the eight algorithms. It can be seen that the HSB-GWO wins or draws with seven comparative algorithms in 82.76% of optimization tasks. Therefore, our proposed algorithm exhibited a favorable balance between exploration and exploitation, as the results reveal, which illustrates the high-performing search ability on complex optimization tasks.

#### 4.2.2. Results of IEEE CEC 2019

To comprehensively demonstrate the performance of our proposed HSB-GWO, the benchmark functions of IEEE CEC 2019 were determined. The results are summarized in Table 7. For F1, both HSB-GWO and CBOA can find the optimal solution for all the trials. Although AGWO and CBOA obtained the best results in F2 and F3, respectively, HSB-GWO performed competitively, being the second-best in F2 and F3. For F4–F10, HSB-GWO outperformed the other seven algorithms, demonstrating a strong search ability. The rank results based on the Wilcoxon signed-rank test further verified the superiority of HSB-GWO because it achieved seven wins, one tie, and two losses in the IEEE CEC 2019 benchmark suite.

**Remark** **5.** *In addition to verifying the balance of exploitation and exploration behaviors through the experimental results, it can also be measured indirectly [47,48] (diversity-based, entropy-based, fitness-based) or directly [49] (attraction basin-based).*

#### 4.2.3. Statistical Analysis by Non-Parametric Friedman Test

To comprehensively evaluate the performance of the HSB-GWO against other algorithms, the Friedman test proposed in [38] is adopted, and the results are presented in Table 8. First, observing the “Overall Rank”, HSB-GWO achieved the top rank (rank 1) in all dimensions (D=10,30,50). In contrast, other algorithms have lower overall rankings or show inconsistent performance in different dimensions. Analyzing the “Avg. Rank”, HSB-GWO has the lowest average rank among all algorithms for each dimension. When D=10, its average rank is 1.66; for D=30, it is 1.17; and for D=50, the average rank of HSB-GWO is 1.14. Lower average ranks signify better overall performance in the Friedman test, which evaluates the relative performance of algorithms in 29 test functions. Moreover, examining the performance on individual test functions (F1, F3–F30) across different dimensions, HSB-GWO consistently delivers optimal or near-optimal results. For example, HSB-GWO obtained the lowest scores compared to other algorithms on F3–F7, F9, F11–F15, F22, F26, and F29 in three different dimensions (D=10,30,50). Moreover, in other test functions like F10 (D=10,30,50), F17 (D=30), and F23 (D=30), HSB-GWO also achieved competitive rankings compared to the best. In summary, the Friedman test results clearly demonstrate that HSB-GWO outperformed other comparative algorithms in terms of both exploration and exploitation capabilities, exhibiting more excellent search performance.

The Friedman test results of the eight algorithms in IEEE CEC 2019 are shown in Table 9. HSB-GWO achieved the best performance, as the average rank is only 1.17, indicating that HSB-GWO ranked high in most test functions. For example, HSB-GWO obtained the lowest scores in F4, F5, F6, F7, F9, and F10. In contrast, other algorithms obtained lower or inconsistent average ranks in the benchmark functions. For example, AOA received an average rank of 2.8 in F1. However, it obtained 7.05 average ranks in F2 and F6, which demonstrates the unstable search performance of AOA.

**Figure 5:**
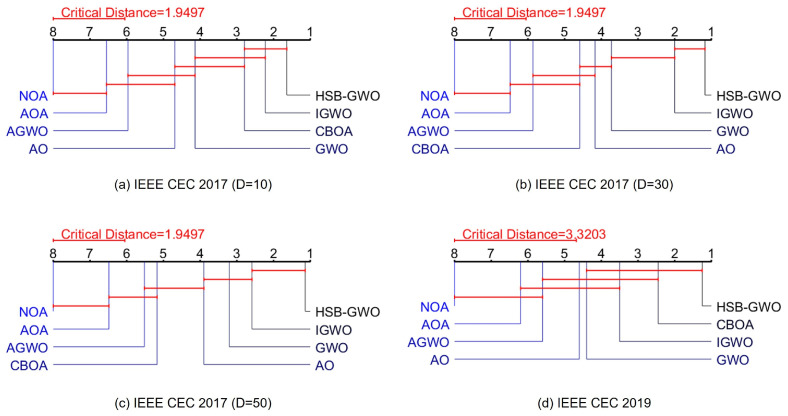
Critical difference (CD) results for the Friedman test of IEEE CEC 2017 and 2019.

Moreover, Figure 5 shows the results of the critical difference (CD) in the Friedman test (Table 8 and Table 9). For D=10 of IEEE CEC 2017, the high performance of HSB-GWO is statistically significant compared to the other algorithms except IGWO. For D=30 and 50 of IEEE CEC 2017, the superiority of HSB-GWO on the search performance is statistically significant. Figure 5d suggests that HSB-GWO significantly outperforms AO, AGWO, AOA, and NOA in IEEE CEC 2019.

### 4.3. Cooperative Path Planning for Multiple UAVs

Eight algorithms were adopted in the two cooperative path planning tasks for multiple UAVs. For all algorithms, we conducted 10 trials, and the maximum number of fitness evaluations (MaxFEs) was set to 2400, and the value of population size $N_{p}=60$. The parameter setting and threat design regarding the cooperative path planning simulation are summarized in Table 10, Table 11, Table 12 and Table 13.

#### 4.3.1. Task 1

Table 14 summarizes the fitness results of eight algorithms after we separately performed 10 trials in **Task 1** for the cooperative path planning of multiple UAVs. HSB-GWO achieved the lowest mean value of 30.81 among all algorithms, which indicates the better performance of HSB-GWO in path planning, as it implies a more optimal path with shorter distance or lower cost. Through the *p*-value results, it can be seen that the performance improvement of HSB-GWO in cooperative path planning tasks for multiple UAVs is statistically different from other compared algorithms. Therefore, the superiority of HSB-GWO is demonstrated. The distances, flight time, velocity, and collision of each UAV in the best trial obtained by each algorithm are provided in Table 15.

**Figure 6:**
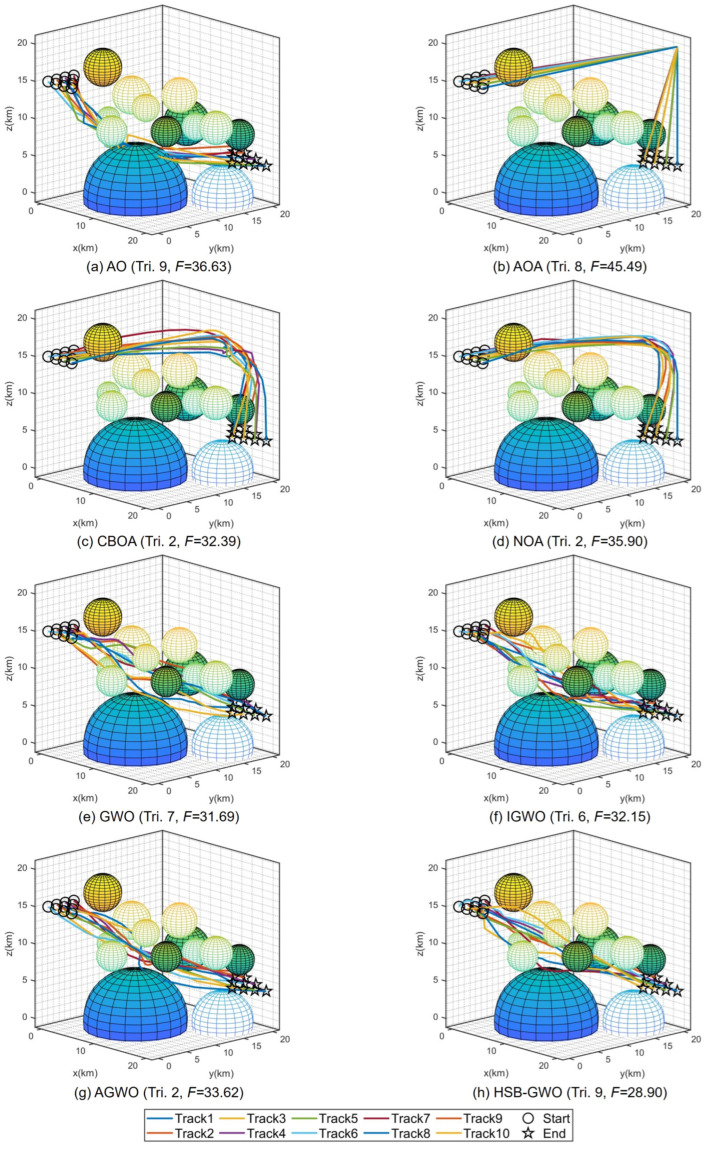
Three-dimensional diagrams of the optimal trial results of 8 algorithms for Task 1.

**Figure 7:**
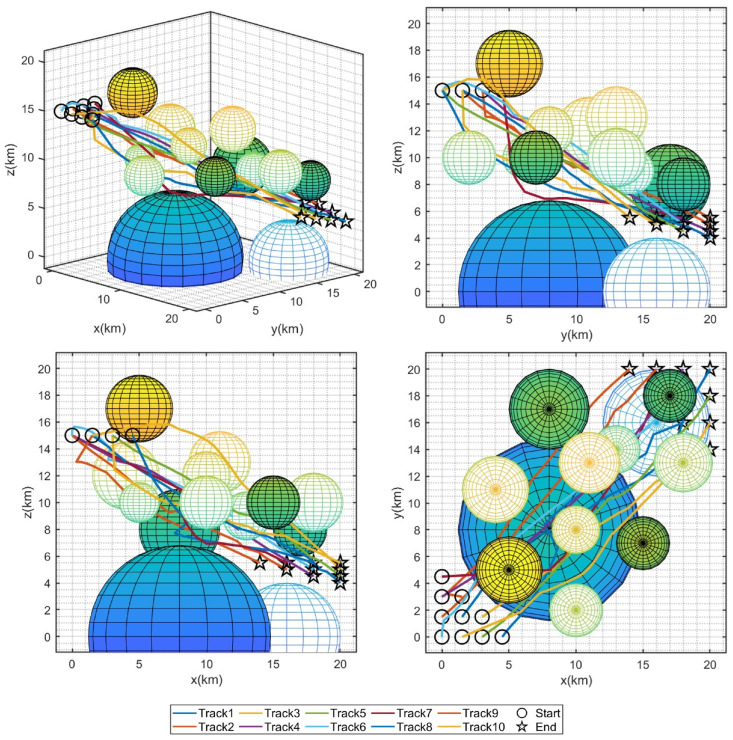
Three views and 3D graph of the best trial result of HSB-GWO for Task 1.

Figure 6 presents the 3D flight trajectory maps of multiple UAVs planned by each algorithm, where the trajectory corresponding to the best trial (the trial boxed in Table 14 with the lowest fitness value) of each algorithm is selected for representation. It can be seen that multiple UAVs flying in a straight line as much as possible from the starting point along the target point is a potential optimal path because the flight distance is shorter, thereby decreasing the flight time and saving more energy. However, this path passes through multiple threat areas, and the algorithms need to guide the UAVs to avoid entering the threat area as much as possible while satisfying the constraints of time and space coordination, which increases the search difficulty of the algorithms. Therefore, AOA, CBOA, and NOA tended to select paths that bypassed threat dense regions, which reduced the extra cost incurred by traversing threat zones. Nevertheless, such paths led to an increase in flight distance and energy consumption, rendering them locally optimal solutions. For the result obtained by AO, the path is a trade-off solution, which shortens the path in the vicinity of threat dense regions by traversing some threat areas. However, as indicated by the fitness, this path also corresponds to a locally optimal solution. GWO, IGWO, AGWO, and HSB-GWO have discovered paths with similar characteristics, yet from the comparison of their fitness, it is evident that the path determined by HSB-GWO incurs the lowest cost for the multiple UAVs. Three views and a 3D graph of the best trial result for HSB-GWO are shown in Figure 7.

**Figure 8:**
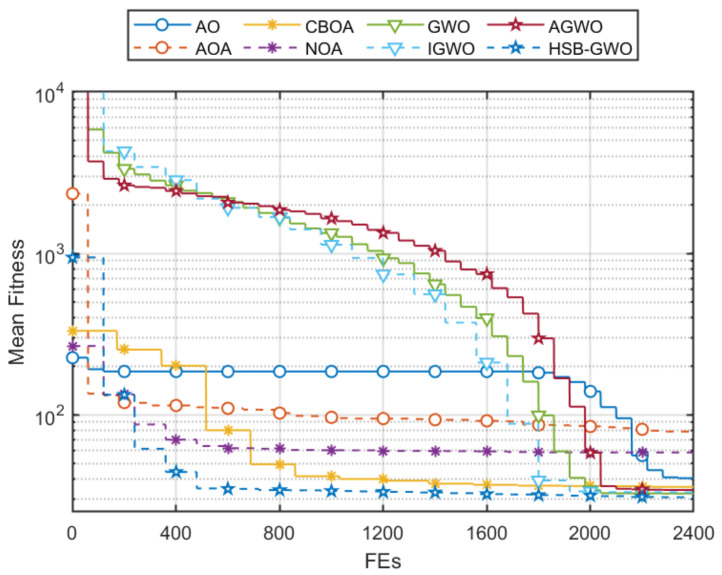
Mean fitness convergences of 8 algorithms in 10 trials for Task 1.

Mean fitness convergences of the eight algorithms are shown in Figure 8. The curves show that HSB-GWO can quickly converge to the local optimal solution in the early stages of iteration and gradually approach the global optimal solution in subsequent searches. For AOA and NOA, their curves showed convergence stagnation during the search process, indicating that their population was trapped in a local optimal solution. For the other comparative algorithms, it can be seen that the fitness stagnation occurred in the middle stage of iteration and converged again in the later stage. The similar convergence characteristics are caused by their hybrid search strategies of dynamical adjustment of exploitation and exploration search behavior. Although these strategies can help the population escape from local optima, the problem of convergence stagnation has not been solved.

#### 4.3.2. Task 2

For **Task 2**, the fitness results are summarized in Table 16. It can be seen that HSB-GWO achieved the lowest mean value of 2.11 compared to other algorithms, indicating the best performance of HSB-GWO in path planning. The results of *p*-values verified that the performance improvement of HSB-GWO is statistically different from other comparative algorithms. Therefore, the superiority of HSB-GWO is demonstrated. The distances, flight time, velocity, and collision of each UAV in the best trial of each algorithm are provided in Table 17.

**Figure 9:**
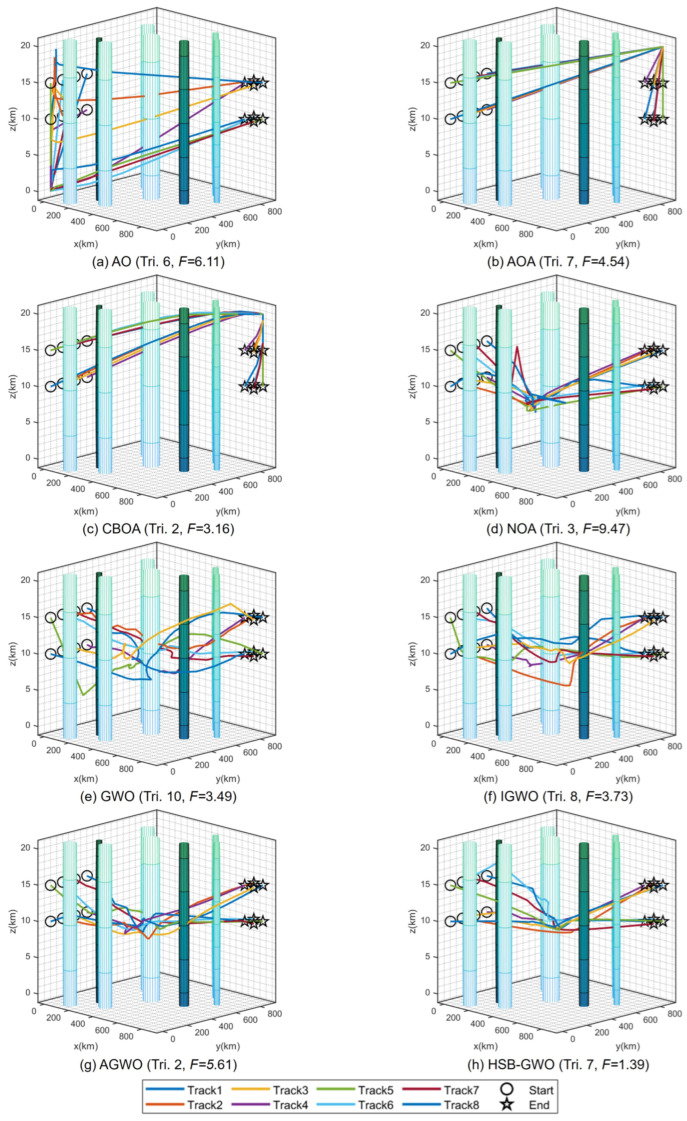
Three-dimensional diagrams of the optimal trial results of 8 algorithms for Task 2.

Figure 9 presents the best flight trajectories of multiple UAVs planned by each algorithm. In **Task 2**, the UAVs need to depart from the initial point and bypass the threat zones to reach the goal positions. Due to the fact that UAVs above the queue ($z_{0}=15$) need to reach the target point at the bottom ($z_{G}=10$), and UAVs departing from below ($z_{0}=10$) need to reach the endpoint above ($z_{G}=15$), they need to avoid collision issues during queue position exchange as much as possible, which increases the difficulty of the path planning.

**Figure 10:**
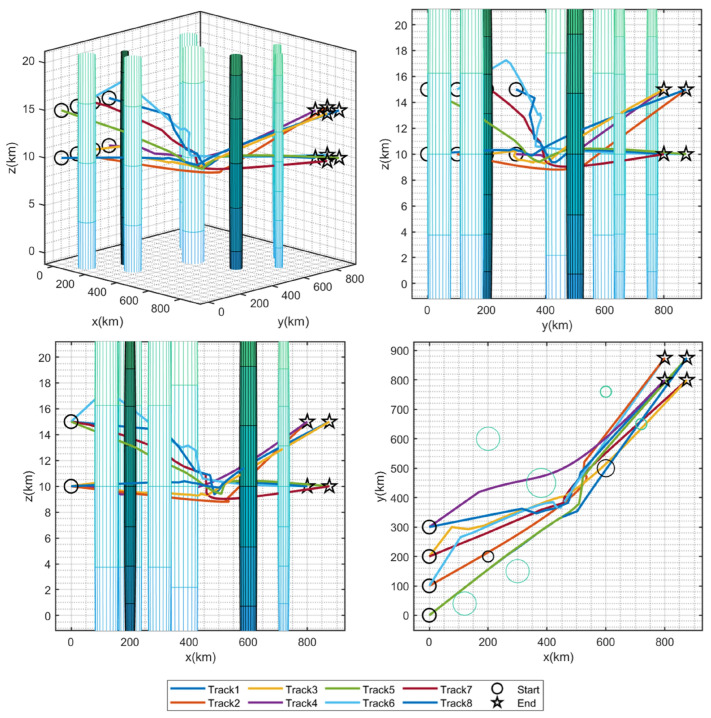
Three views and 3D graph of the best trial result of HSB-GWO for Task 2.

It can be seen that the strategies adopted by the eight algorithms were different. For example, AO reduced the collisions by implementing a strategy of exchanging positions of UAVs from the starting positions. For AOA and CBOA, similar paths were generated that increased the difference in flight distance between the upper and lower UAVs to reduce the possibility of collision due to the time difference. However, both strategies were considered as local optimal paths due to increased flight distances and energy consumption. The other five algorithms adopted the same strategy, which is to achieve position exchange while moving towards the target positions. However, the conservative routes designed by GWO and IGWO were adopted for some UAVs via a detour to decrease the possibility of collisions. Conversely, AGWO and HSB-GWO achieved coordinated flight by setting different flight velocities for UAVs. Three views and a 3D graph of the best trial result for HSB-GWO are shown in Figure 10.

**Figure 11:**
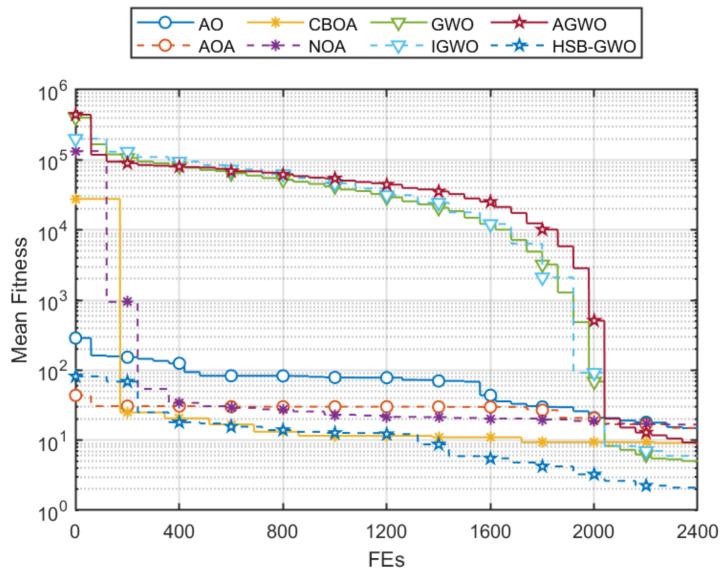
Mean fitness convergences of 8 algorithms in 10 trials for Task 2.

Mean fitness convergences of the eight algorithms are shown in Figure 11, which shows that HSB-GWO exhibited remarkable convergence behavior because the HSB-GWO quickly reduced its mean fitness in the early stage of optimization and converged to the lowest fitness when the search finished. It is worth noting that during the search process, AGWO, IGWO, and GWO converged slowly for a period of time. Although the algorithm ultimately found a better solution, this indicates that there are shortcomings in the algorithm. Although AOA, CBOA, and NOA achieved a rapid decrease in average fitness in the early stages of search, they fell into local optima after FEs=400.

### 4.4. Architecture Effectiveness Analysis of HSB-GWO

**Figure 12:**
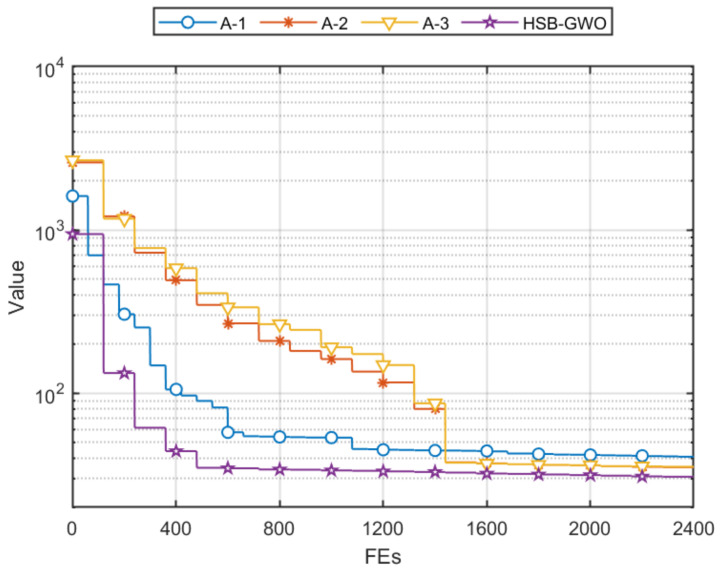
Mean fitness convergences of three ablation algorithms and HSB-GWO in 10 trials using Task 1.

The ablation simulation was conducted to verify the architecture effectiveness of the proposed HSB-GWO. The simulation configuration is the same as Section 4.3 and three ablation algorithms are designed: HSB-GWO without DLH (A-1), HSB-GWO without Aquila exploration (A-2), and HSB-GWO without adaptive search (A-3). The simulation results and mean fitness convergences are shown in Table 18 and Figure 12, respectively. From the curve of A-1, it can be seen that the hybrid search mode composed of Aquila exploration and adaptive search can effectively address the problem of convergence stagnation, but A-1 without DLH cannot further approach the global optimal solution, whereby the mean fitness is 40.86. Conversely, the curves of A-2 and A-3 indicate that the fitness convergence stagnation is unavoidable, which affects search efficiency. In addition, the *p*-values represented in Table 18 of the three ablation algorithms demonstrate that the designed architecture has a statistically significant effect on improving search performance of the HSB-GWO.

## 5. Conclusions

This study proposes a hybrid search behavior-based adaptive grey wolf optimizer (HSB-GWO) to address the limitations of traditional metaheuristic algorithms in complex optimization tasks and multi-UAV cooperative path planning. Through theoretical analysis, benchmark function experiments, and multi-UAV path planning simulations, the effectiveness and superiority of HSB-GWO are systematically verified. According to the results on optimization for the benchmark functions, HSB-GWO exhibits excellent search capabilities across different types of optimization tasks. On the IEEE CEC 2017 benchmark suite (including unimodal, multimodal, hybrid, and composite functions), HSB-GWO outperformed seven metaheuristic algorithms (AO, AOA, CBOA, NOA, GWO, IGWO, and AGWO). Ablation experiments show that the three core components of HSB-GWO (DLH, Aquila exploration, and adaptive search) play crucial roles in improving search performance, in which DLH enhances the search ability of algorithm and hybrid search mode (Aquila exploration and adaptive search) effectively avoids premature convergence and enhances the ability to escape local optima. Statistical analysis confirms that the performance improvement of HSB-GWO is statistically significant. In the multi-UAV cooperative path planning scenario with 10 UAVs and 13 threats, HSB-GWO achieves the lowest mean fitness (30.81) among all algorithms. Additionally, HSB-GWO’s convergence curve shows fast early convergence and stable late-stage optimization, avoiding the convergence stagnation problems.

## 6. Future Work

In this work, HSB-GWO was proposed for global optimal path planning. However, in practical applications, in order to better cope with environmental changes, a hybrid architecture of global and local path planning is adopted to achieve collaborative control of UAVs. In the design of local path planning algorithms, it is necessary to consider issues such as slow communication speed, limited sensors, or restrictions on UAV movement due to external interference. Combining sensor data and control technology is an effective solution for designing efficient local path planning algorithms [50,51,52]. Therefore, in future work, we will further introduce relevant control techniques based on the existing HSB-GWO to implement a hybrid (global + local) path planning algorithm, improving the algorithm’s adaptability to dynamic obstacles and unexpected situations.

## Figures and Tables

**Table 1:** Mean ranks of HSB-GWOs with different control parameter settings using IEEE CEC 2017.

F	D	$r_{E}=0.2$	$r_{E}=0.3$	$r_{E}=0.4$	$r_{E}=0.5$	$r_{E}=0.6$	$r_{E}=0.7$	$r_{E}=0.8$
F1, F3	10	**1.00**	2.00	3.50	3.50	5.50	5.50	7.00
	30	2.00	5.50	5.50	**1.50**	2.50	6.00	5.00
	50	**1.50**	2.00	3.00	5.50	5.50	4.50	6.00
F4–F10	10	**1.57**	2.57	2.86	3.14	5.00	6.29	6.57
	30	**1.43**	1.57	4.00	3.86	5.00	6.14	6.00
	50	**2.29**	2.43	3.86	3.43	4.43	5.71	5.86
F11–F20	10	3.20	3.20	3.90	**2.60**	4.30	4.90	5.90
	30	3.30	**2.90**	4.10	3.20	4.70	5.20	4.60
	50	**2.40**	3.50	4.00	4.50	4.40	4.50	4.70
F21–F30	10	3.30	4.00	3.90	3.50	**3.20**	4.70	5.40
	30	**2.20**	2.70	4.30	3.30	4.00	5.40	6.10
	50	**3.20**	3.40	3.70	4.00	4.30	5.10	4.30
Overall	10	**2.69**	3.24	3.62	3.10	4.17	5.21	5.97
	30	**2.38**	2.69	4.24	3.28	4.38	5.55	5.48
	50	**2.59**	3.10	3.79	4.14	4.45	5.00	4.93

**Bold font** denotes the best result.

**Table 2:** The comparison of obtained solutions for unimodal functions of IEEE CEC 2017.

F	D	Index	Algorithm							
			AO (2025)	AOA (2021)	CBOA (2022)	NOA (2023)	GWO (2024)	IGWO (2021)	AGWO (2022)	HSB-GWO
F1	10	MFE	2.10 $\times \;10^{5}$	2.13 $\times \;10^{9}$	$1.21\times 10^{2}$	1.04 $\times \;10^{10}$	2.49 $\times \;10^{6}$	3.48 $\times \;10^{4}$	1.24 $\times \;10^{9}$	8.25 $\times \;10^{3}$
		STD	1.16 $\times \;10^{5}$	1.28 $\times \;10^{9}$	1.70 $\times \;10^{2}$	2.96 $\times \;10^{9}$	7.09 $\times \;10^{6}$	1.62 $\times \;10^{4}$	8.84 $\times \;10^{8}$	3.96 $\times \;10^{3}$
	30	MFE	3.17 $\times \;10^{6}$	4.05 $\times \;10^{10}$	1.02 $\times \;10^{10}$	8.15 $\times \;10^{10}$	8.77 $\times \;10^{8}$	3.17 $\times \;10^{5}$	4.95 $\times \;10^{9}$	$3.51\times 10^{3}$
		STD	1.22 $\times \;10^{6}$	4.69 $\times \;10^{9}$	2.14 $\times \;10^{9}$	7.82 $\times \;10^{9}$	9.14 $\times \;10^{8}$	1.79 $\times \;10^{5}$	3.06 $\times \;10^{9}$	3.30 $\times \;10^{3}$
	50	MFE	1.21 $\times \;10^{7}$	1.05 $\times \;10^{11}$	4.90 $\times \;10^{10}$	1.84 $\times \;10^{11}$	4.48 $\times \;10^{9}$	$3.46\times 10^{3}$	1.17 $\times \;10^{10}$	7.62 $\times \;10^{3}$
		STD	2.60 $\times \;10^{6}$	8.26 $\times \;10^{9}$	6.29 $\times \;10^{9}$	1.57 $\times \;10^{10}$	2.02 $\times \;10^{9}$	2.48 $\times \;10^{3}$	3.84 $\times \;10^{9}$	1.00 $\times \;10^{4}$
F3	10	MFE	3.97 $\times \;10^{0}$	4.79 $\times \;10^{3}$	7.50 $\times \;10^{2}$	2.89 $\times \;10^{4}$	4.25 $\times \;10^{2}$	2.33 $\times \;10^{-1}$	2.54 $\times \;10^{3}$	$7.81\times 10^{-7}$
		STD	3.87 $\times \;10^{0}$	2.93 $\times \;10^{3}$	4.19 $\times \;10^{2}$	1.14 $\times \;10^{4}$	9.18 $\times \;10^{2}$	1.64 $\times \;10^{-1}$	2.29 $\times \;10^{3}$	1.50 $\times \;10^{-6}$
	30	MFE	6.67 $\times \;10^{3}$	7.21 $\times \;10^{4}$	5.56 $\times \;10^{4}$	2.14 $\times \;10^{5}$	3.33 $\times \;10^{4}$	1.49 $\times \;10^{3}$	4.79 $\times \;10^{4}$	$8.57\times 10^{2}$
		STD	2.06 $\times \;10^{3}$	8.13 $\times \;10^{3}$	6.13 $\times \;10^{3}$	3.47 $\times \;10^{4}$	8.97 $\times \;10^{3}$	1.07 $\times \;10^{3}$	9.37 $\times \;10^{3}$	9.86 $\times \;10^{2}$
	50	MFE	5.50 $\times \;10^{4}$	1.63 $\times \;10^{5}$	1.26 $\times \;10^{5}$	3.67 $\times \;10^{5}$	7.08 $\times \;10^{4}$	1.01 $\times \;10^{4}$	9.05 $\times \;10^{4}$	$4.68\times 10^{3}$
		STD	1.20 $\times \;10^{4}$	1.86 $\times \;10^{4}$	1.11 $\times \;10^{4}$	6.72 $\times \;10^{4}$	1.38 $\times \;10^{4}$	3.35 $\times \;10^{3}$	1.97 $\times \;10^{4}$	3.60 $\times \;10^{3}$
Rank	10	w/t/l	0/0/2	0/0/2	1/0/1	0/0/2	0/0/2	0/0/2	0/0/2	1/0/1
	30	w/t/l	0/0/2	0/0/2	0/0/2	0/0/2	0/0/2	1/0/1	0/0/2	2/0/0
	50	w/t/l	0/0/2	0/0/2	0/0/2	0/0/2	0/0/2	0/0/2	0/0/2	1/0/1

**Bold font** denotes the best result.

**Table 3:** The comparison of obtained solutions for multimodal functions of IEEE CEC 2017.

F	D	Index	Algorithm							
			AO (2025)	AOA (2021)	CBOA (2022)	NOA (2023)	GWO (2024)	IGWO (2021)	AGWO (2022)	HSB-GWO
F4	10	MFE	8.32 $\times \;10^{0}$	1.17 $\times \;10^{2}$	7.75 $\times \;10^{0}$	7.84 $\times \;10^{2}$	1.03 $\times \;10^{1}$	3.10 $\times \;10^{0}$	6.82 $\times \;10^{1}$	$2.23\times 10^{-2}$
		STD	1.37 $\times \;10^{1}$	7.74 $\times \;10^{1}$	1.08 $\times \;10^{1}$	1.91 $\times \;10^{2}$	1.28 $\times \;10^{1}$	5.31 $\times \;10^{-1}$	3.51 $\times \;10^{1}$	7.92 $\times \;10^{-3}$
	30	MFE	1.19 $\times \;10^{2}$	8.60 $\times \;10^{3}$	2.18 $\times \;10^{3}$	2.27 $\times \;10^{4}$	1.41 $\times \;10^{2}$	8.96 $\times \;10^{1}$	5.27 $\times \;10^{2}$	$7.51\times 10^{1}$
		STD	2.02 $\times \;10^{1}$	2.47 $\times \;10^{3}$	5.22 $\times \;10^{2}$	4.14 $\times \;10^{3}$	3.74 $\times \;10^{1}$	1.71 $\times \;10^{0}$	7.41 $\times \;10^{2}$	2.32 $\times \;10^{1}$
	50	MFE	2.18 $\times \;10^{2}$	2.61 $\times \;10^{4}$	1.05 $\times \;10^{4}$	5.94 $\times \;10^{4}$	4.04 $\times \;10^{2}$	1.34 $\times \;10^{2}$	1.82 $\times \;10^{3}$	$7.09\times 10^{1}$
		STD	6.00 $\times \;10^{1}$	5.09 $\times \;10^{3}$	2.32 $\times \;10^{3}$	1.16 $\times \;10^{4}$	1.14 $\times \;10^{2}$	4.89 $\times \;10^{1}$	9.02 $\times \;10^{2}$	5.85 $\times \;10^{1}$
F5	10	MFE	2.26 $\times \;10^{1}$	4.72 $\times \;10^{1}$	1.07 $\times \;10^{1}$	1.07 $\times \;10^{2}$	1.24 $\times \;10^{1}$	1.27 $\times \;10^{1}$	4.91 $\times \;10^{1}$	$6.93\times 10^{0}$
		STD	7.91 $\times \;10^{0}$	1.52 $\times \;10^{1}$	4.03 $\times \;10^{0}$	2.03 $\times \;10^{1}$	4.97 $\times \;10^{0}$	6.64 $\times \;10^{0}$	6.94 $\times \;10^{0}$	2.75 $\times \;10^{0}$
	30	MFE	1.48 $\times \;10^{2}$	2.96 $\times \;10^{2}$	1.45 $\times \;10^{2}$	5.37 $\times \;10^{2}$	9.67 $\times \;10^{1}$	5.22 $\times \;10^{1}$	2.17 $\times \;10^{2}$	$3.71\times 10^{1}$
		STD	2.85 $\times \;10^{1}$	3.83 $\times \;10^{1}$	3.20 $\times \;10^{1}$	2.64 $\times \;10^{1}$	4.22 $\times \;10^{1}$	3.72 $\times \;10^{1}$	4.24 $\times \;10^{1}$	1.05 $\times \;10^{1}$
	50	MFE	2.99 $\times \;10^{2}$	5.74 $\times \;10^{2}$	2.89 $\times \;10^{2}$	9.86 $\times \;10^{2}$	1.80 $\times \;10^{2}$	2.07 $\times \;10^{2}$	3.89 $\times \;10^{2}$	$1.15\times 10^{2}$
		STD	3.52 $\times \;10^{1}$	3.77 $\times \;10^{1}$	2.95 $\times \;10^{1}$	5.32 $\times \;10^{1}$	2.87 $\times \;10^{1}$	5.37 $\times \;10^{1}$	5.41 $\times \;10^{1}$	4.59 $\times \;10^{1}$
F6	10	MFE	1.19 $\times \;10^{1}$	3.43 $\times \;10^{1}$	1.34 $\times \;10^{-1}$	6.57 $\times \;10^{1}$	4.25 $\times \;10^{-1}$	9.85 $\times \;10^{-2}$	2.35 $\times \;10^{1}$	$1.34\times 10^{-10}$
		STD	5.40 $\times \;10^{0}$	6.77 $\times \;10^{0}$	1.60 $\times \;10^{-1}$	6.45 $\times \;10^{0}$	6.52 $\times \;10^{-1}$	3.06 $\times \;10^{-2}$	4.98 $\times \;10^{0}$	1.08 $\times \;10^{-10}$
	30	MFE	3.72 $\times \;10^{1}$	6.19 $\times \;10^{1}$	6.87 $\times \;10^{0}$	1.08 $\times \;10^{2}$	4.73 $\times \;10^{0}$	2.18 $\times \;10^{-1}$	4.33 $\times \;10^{1}$	$3.46\times 10^{-2}$
		STD	7.36 $\times \;10^{0}$	5.66 $\times \;10^{0}$	3.67 $\times \;10^{0}$	7.39 $\times \;10^{0}$	3.78 $\times \;10^{0}$	7.51 $\times \;10^{-2}$	8.12 $\times \;10^{0}$	1.07 $\times \;10^{-1}$
	50	MFE	4.92 $\times \;10^{1}$	8.22 $\times \;10^{1}$	1.50 $\times \;10^{1}$	1.27 $\times \;10^{2}$	1.05 $\times \;10^{1}$	2.73 $\times \;10^{-1}$	5.74 $\times \;10^{1}$	$2.92\times 10^{-2}$
		STD	7.39 $\times \;10^{0}$	5.92 $\times \;10^{0}$	3.95 $\times \;10^{0}$	5.10 $\times \;10^{0}$	3.38 $\times \;10^{0}$	8.10 $\times \;10^{-2}$	8.16 $\times \;10^{0}$	5.22 $\times \;10^{-2}$
F7	10	MFE	5.24 $\times \;10^{1}$	9.09 $\times \;10^{1}$	1.99 $\times \;10^{1}$	2.97 $\times \;10^{2}$	2.46 $\times \;10^{1}$	2.28 $\times \;10^{1}$	7.44 $\times \;10^{1}$	$1.66\times 10^{1}$
		STD	1.73 $\times \;10^{1}$	1.88 $\times \;10^{1}$	3.44 $\times \;10^{0}$	3.95 $\times \;10^{1}$	8.63 $\times \;10^{0}$	9.46 $\times \;10^{0}$	8.31 $\times \;10^{0}$	3.25 $\times \;10^{0}$
	30	MFE	2.59 $\times \;10^{2}$	5.91 $\times \;10^{2}$	2.70 $\times \;10^{2}$	2.04 $\times \;10^{3}$	1.25 $\times \;10^{2}$	1.04 $\times \;10^{2}$	3.88 $\times \;10^{2}$	$6.63\times 10^{1}$
		STD	3.36 $\times \;10^{1}$	6.18 $\times \;10^{1}$	3.40 $\times \;10^{1}$	1.17 $\times \;10^{2}$	2.87 $\times \;10^{1}$	5.49 $\times \;10^{1}$	9.18 $\times \;10^{1}$	1.50 $\times \;10^{1}$
	50	MFE	5.69 $\times \;10^{2}$	1.14 $\times \;10^{3}$	6.28 $\times \;10^{2}$	4.25 $\times \;10^{3}$	3.17 $\times \;10^{2}$	4.06 $\times \;10^{2}$	8.44 $\times \;10^{2}$	$2.66\times 10^{2}$
		STD	1.02 $\times \;10^{2}$	8.00 $\times \;10^{1}$	7.23 $\times \;10^{1}$	2.12 $\times \;10^{2}$	6.83 $\times \;10^{1}$	1.92 $\times \;10^{1}$	1.40 $\times \;10^{2}$	2.93 $\times \;10^{2}$
F8	10	MFE	2.03 $\times \;10^{1}$	2.93 $\times \;10^{1}$	7.12 $\times \;10^{0}$	1.07 $\times \;10^{2}$	1.06 $\times \;10^{1}$	8.33 $\times \;10^{0}$	2.99 $\times \;10^{1}$	$5.07\times 10^{0}$
		STD	6.89 $\times \;10^{0}$	6.49 $\times \;10^{0}$	2.35 $\times \;10^{0}$	1.13 $\times \;10^{1}$	4.16 $\times \;10^{0}$	6.74 $\times \;10^{0}$	6.31 $\times \;10^{0}$	2.39 $\times \;10^{0}$
	30	MFE	1.34 $\times \;10^{2}$	2.45 $\times \;10^{2}$	1.04 $\times \;10^{2}$	4.94 $\times \;10^{2}$	7.04 $\times \;10^{1}$	$4.17\times 10^{1}$	1.58 $\times \;10^{2}$	4.33 $\times \;10^{1}$
		STD	1.77 $\times \;10^{1}$	3.02 $\times \;10^{1}$	1.47 $\times \;10^{1}$	2.33 $\times \;10^{1}$	2.34 $\times \;10^{1}$	3.05 $\times \;10^{1}$	3.67 $\times \;10^{1}$	2.01 $\times \;10^{1}$
	50	MFE	3.20 $\times \;10^{2}$	6.07 $\times \;10^{2}$	2.83 $\times \;10^{2}$	9.77 $\times \;10^{2}$	1.94 $\times \;10^{2}$	2.68 $\times \;10^{2}$	3.66 $\times \;10^{2}$	$9.61\times 10^{1}$
		STD	3.66 $\times \;10^{1}$	2.19 $\times \;10^{1}$	3.37 $\times \;10^{1}$	5.14 $\times \;10^{1}$	3.97 $\times \;10^{1}$	4.16 $\times \;10^{1}$	5.38 $\times \;10^{1}$	3.86 $\times \;10^{1}$
F9	10	MFE	5.55 $\times \;10^{1}$	4.09 $\times \;10^{2}$	3.81 $\times \;10^{0}$	2.31 $\times \;10^{3}$	6.72 $\times \;10^{0}$	1.63 $\times \;10^{-2}$	1.20 $\times \;10^{2}$	$0.00\times 10^{0}$
		STD	6.01 $\times \;10^{1}$	2.31 $\times \;10^{2}$	7.33 $\times \;10^{0}$	5.53 $\times \;10^{2}$	1.19 $\times \;10^{1}$	1.16 $\times \;10^{-2}$	6.41 $\times \;10^{1}$	0.00 $\times \;10^{0}$
	30	MFE	3.92 $\times \;10^{3}$	4.62 $\times \;10^{3}$	1.64 $\times \;10^{3}$	2.24 $\times \;10^{4}$	4.29 $\times \;10^{2}$	7.90 $\times \;10^{-1}$	5.14 $\times \;10^{3}$	$0.00\times 10^{0}$
		STD	1.13 $\times \;10^{3}$	5.86 $\times \;10^{2}$	2.61 $\times \;10^{2}$	2.96 $\times \;10^{3}$	3.18 $\times \;10^{2}$	4.26 $\times \;10^{-1}$	1.60 $\times \;10^{3}$	0.00 $\times \;10^{0}$
	50	MFE	1.32 $\times \;10^{4}$	2.28 $\times \;10^{4}$	8.01 $\times \;10^{3}$	6.52 $\times \;10^{4}$	3.90 $\times \;10^{3}$	1.20 $\times \;10^{1}$	2.16 $\times \;10^{4}$	$2.72\times 10^{-2}$
		STD	2.60 $\times \;10^{3}$	2.64 $\times \;10^{3}$	9.33 $\times \;10^{2}$	6.36 $\times \;10^{3}$	2.48 $\times \;10^{3}$	1.35 $\times \;10^{1}$	5.58 $\times \;10^{3}$	1.03 $\times \;10^{-1}$
F10	10	MFE	7.41 $\times \;10^{2}$	1.04 $\times \;10^{3}$	$1.69\times 10^{2}$	2.11 $\times \;10^{3}$	4.92 $\times \;10^{2}$	5.54 $\times \;10^{2}$	1.10 $\times \;10^{3}$	2.26 $\times \;10^{2}$
		STD	2.63 $\times \;10^{2}$	2.26 $\times \;10^{2}$	1.02 $\times \;10^{2}$	2.94 $\times \;10^{2}$	3.00 $\times \;10^{2}$	4.58 $\times \;10^{2}$	2.61 $\times \;10^{2}$	2.17 $\times \;10^{2}$
	30	MFE	3.96 $\times \;10^{3}$	5.24 $\times \;10^{3}$	$2.22\times 10^{3}$	8.39 $\times \;10^{3}$	3.08 $\times \;10^{3}$	3.78 $\times \;10^{3}$	4.66 $\times \;10^{3}$	2.41 $\times \;10^{3}$
		STD	5.58 $\times \;10^{2}$	5.43 $\times \;10^{2}$	2.95 $\times \;10^{2}$	3.14 $\times \;10^{2}$	1.27 $\times \;10^{3}$	2.53 $\times \;10^{3}$	6.13 $\times \;10^{2}$	9.06 $\times \;10^{2}$
	50	MFE	6.43 $\times \;10^{3}$	1.12 $\times \;10^{4}$	$4.41\times 10^{3}$	1.50 $\times \;10^{4}$	5.97 $\times \;10^{3}$	9.72 $\times \;10^{3}$	8.62 $\times \;10^{3}$	4.70 $\times \;10^{3}$
		STD	7.36 $\times \;10^{2}$	7.22 $\times \;10^{2}$	6.47 $\times \;10^{2}$	4.58 $\times \;10^{2}$	1.95 $\times \;10^{3}$	2.53 $\times \;10^{3}$	1.10 $\times \;10^{3}$	1.13 $\times \;10^{3}$
Rank	10	w/t/l	0/0/7	0/0/7	1/0/6	0/0/7	0/0/7	0/0/7	0/0/7	6/0/1
	30	w/t/l	0/0/7	0/0/7	1/0/6	0/0/7	0/0/7	1/0/6	0/0/7	5/0/2
	50	w/t/l	0/0/7	0/0/7	1/0/6	0/0/7	0/0/7	0/0/7	0/0/7	6/0/1

**Bold font** denotes the best result.

**Table 4:** The comparison of obtained solutions for hybrid functions of IEEE CEC 2017.

F	D	Index	Algorithm							
			AO (2025)	AOA (2021)	CBOA (2022)	NOA (2023)	GWO (2024)	IGWO (2021)	AGWO (2022)	HSB-GWO
F11	10	MFE	5.80 $\times \;10^{1}$	4.73 $\times \;10^{1}$	1.17 $\times \;10^{1}$	3.21 $\times \;10^{3}$	2.50 $\times \;10^{1}$	3.51 $\times \;10^{0}$	2.96 $\times \;10^{2}$	$1.35\times 10^{0}$
		STD	4.75 $\times \;10^{1}$	2.83 $\times \;10^{1}$	4.62 $\times \;10^{0}$	2.58 $\times \;10^{3}$	2.71 $\times \;10^{1}$	1.44 $\times \;10^{0}$	9.80 $\times \;10^{2}$	1.08 $\times \;10^{0}$
	30	MFE	2.63 $\times \;10^{2}$	2.47 $\times \;10^{3}$	7.54 $\times \;10^{2}$	1.46 $\times \;10^{4}$	2.21 $\times \;10^{2}$	6.41 $\times \;10^{1}$	1.14 $\times \;10^{3}$	$2.41\times 10^{1}$
		STD	6.37 $\times \;10^{1}$	1.63 $\times \;10^{3}$	3.30 $\times \;10^{2}$	3.75 $\times \;10^{3}$	4.81 $\times \;10^{1}$	2.79 $\times \;10^{1}$	8.28 $\times \;10^{2}$	2.36 $\times \;10^{1}$
	50	MFE	3.33 $\times \;10^{2}$	1.40 $\times \;10^{4}$	7.13 $\times \;10^{3}$	4.09 $\times \;10^{4}$	2.23 $\times \;10^{3}$	2.71 $\times \;10^{2}$	4.72 $\times \;10^{3}$	$4.39\times 10^{1}$
		STD	9.10 $\times \;10^{1}$	2.96 $\times \;10^{3}$	8.85 $\times \;10^{2}$	4.19 $\times \;10^{3}$	1.25 $\times \;10^{3}$	2.72 $\times \;10^{1}$	2.75 $\times \;10^{3}$	1.35 $\times \;10^{1}$
F12	10	MFE	2.82 $\times \;10^{6}$	1.25 $\times \;10^{6}$	7.26 $\times \;10^{5}$	5.81 $\times \;10^{8}$	6.12 $\times \;10^{5}$	1.92 $\times \;10^{4}$	3.26 $\times \;10^{6}$	$1.84\times 10^{4}$
		STD	2.56 $\times \;10^{6}$	1.63 $\times \;10^{6}$	7.31 $\times \;10^{5}$	3.19 $\times \;10^{8}$	7.49 $\times \;10^{5}$	3.09 $\times \;10^{4}$	2.46 $\times \;10^{6}$	1.93 $\times \;10^{4}$
	30	MFE	1.14 $\times \;10^{7}$	7.45 $\times \;10^{9}$	1.36 $\times \;10^{9}$	1.41 $\times \;10^{10}$	2.85 $\times \;10^{7}$	1.02 $\times \;10^{6}$	2.20 $\times \;10^{8}$	$8.68\times 10^{5}$
		STD	6.61 $\times \;10^{6}$	1.99 $\times \;10^{9}$	8.84 $\times \;10^{8}$	2.61 $\times \;10^{9}$	3.56 $\times \;10^{7}$	8.31 $\times \;10^{5}$	1.90 $\times \;10^{8}$	4.57 $\times \;10^{4}$
	50	MFE	3.94 $\times \;10^{7}$	5.22 $\times \;10^{10}$	2.65 $\times \;10^{10}$	7.28 $\times \;10^{10}$	3.40 $\times \;10^{8}$	3.27 $\times \;10^{7}$	4.18 $\times \;10^{9}$	$1.26\times 10^{6}$
		STD	1.66 $\times \;10^{7}$	1.11 $\times \;10^{10}$	7.13 $\times \;10^{9}$	1.35 $\times \;10^{10}$	3.12 $\times \;10^{8}$	1.84 $\times \;10^{7}$	3.42 $\times \;10^{9}$	9.07 $\times \;10^{5}$
F13	10	MFE	1.11 $\times \;10^{4}$	7.06 $\times \;10^{3}$	3.18 $\times \;10^{3}$	1.96 $\times \;10^{7}$	8.15 $\times \;10^{3}$	$9.17\times 10^{2}$	1.19 $\times \;10^{4}$	1.15 $\times \;10^{3}$
		STD	7.33 $\times \;10^{3}$	5.83 $\times \;10^{3}$	2.45 $\times \;10^{3}$	1.55 $\times \;10^{7}$	4.07 $\times \;10^{3}$	7.06 $\times \;10^{2}$	8.28 $\times \;10^{3}$	2.17 $\times \;10^{3}$
	30	MFE	1.91 $\times \;10^{5}$	3.70 $\times \;10^{4}$	3.21 $\times \;10^{7}$	8.02 $\times \;10^{9}$	1.94 $\times \;10^{7}$	1.15 $\times \;10^{5}$	4.77 $\times \;10^{7}$	$3.09\times 10^{4}$
		STD	8.90 $\times \;10^{4}$	1.49 $\times \;10^{4}$	6.49 $\times \;10^{7}$	2.36 $\times \;10^{9}$	6.51 $\times \;10^{7}$	5.62 $\times \;10^{4}$	9.00 $\times \;10^{7}$	2.49 $\times \;10^{4}$
	50	MFE	4.40 $\times \;10^{5}$	3.55 $\times \;10^{9}$	1.20 $\times \;10^{10}$	3.54 $\times \;10^{10}$	9.40 $\times \;10^{7}$	5.27 $\times \;10^{6}$	3.86 $\times \;10^{8}$	$3.72\times 10^{3}$
		STD	1.69 $\times \;10^{5}$	3.85 $\times \;10^{9}$	6.15 $\times \;10^{9}$	6.90 $\times \;10^{9}$	1.04 $\times \;10^{8}$	5.79 $\times \;10^{6}$	1.13 $\times \;10^{9}$	4.59 $\times \;10^{3}$
F14	10	MFE	2.18 $\times \;10^{2}$	8.42 $\times \;10^{3}$	7.82 $\times \;10^{1}$	3.87 $\times \;10^{4}$	1.05 $\times \;10^{3}$	5.44 $\times \;10^{1}$	1.76 $\times \;10^{3}$	$1.67\times 10^{1}$
		STD	1.42 $\times \;10^{2}$	7.40 $\times \;10^{3}$	9.49 $\times \;10^{1}$	5.94 $\times \;10^{4}$	1.55 $\times \;10^{3}$	9.77 $\times \;10^{0}$	1.62 $\times \;10^{3}$	1.02 $\times \;10^{1}$
	30	MFE	1.44 $\times \;10^{5}$	5.50 $\times \;10^{4}$	5.02 $\times \;10^{5}$	8.67 $\times \;10^{6}$	1.22 $\times \;10^{5}$	3.17 $\times \;10^{3}$	5.79 $\times \;10^{5}$	$2.41\times 10^{3}$
		STD	1.77 $\times \;10^{5}$	5.20 $\times \;10^{4}$	3.57 $\times \;10^{5}$	3.24 $\times \;10^{6}$	2.51 $\times \;10^{5}$	2.94 $\times \;10^{3}$	5.48 $\times \;10^{5}$	1.80 $\times \;10^{3}$
	50	MFE	6.71 $\times \;10^{5}$	4.48 $\times \;10^{5}$	4.27 $\times \;10^{6}$	6.28 $\times \;10^{7}$	2.43 $\times \;10^{5}$	1.02 $\times \;10^{5}$	1.76 $\times \;10^{6}$	$1.87\times 10^{4}$
		STD	3.75 $\times \;10^{5}$	6.34 $\times \;10^{5}$	3.20 $\times \;10^{6}$	3.25 $\times \;10^{7}$	2.25 $\times \;10^{5}$	6.39 $\times \;10^{4}$	1.61 $\times \;10^{6}$	1.39 $\times \;10^{4}$
F15	10	MFE	1.54 $\times \;10^{3}$	9.60 $\times \;10^{3}$	1.22 $\times \;10^{2}$	1.04 $\times \;10^{5}$	1.23 $\times \;10^{3}$	4.15 $\times \;10^{1}$	1.36 $\times \;10^{3}$	$4.68\times 10^{0}$
		STD	1.14 $\times \;10^{3}$	6.17 $\times \;10^{3}$	2.40 $\times \;10^{2}$	1.03 $\times \;10^{5}$	1.45 $\times \;10^{3}$	2.34 $\times \;10^{1}$	9.29 $\times \;10^{2}$	3.79 $\times \;10^{0}$
	30	MFE	5.38 $\times \;10^{4}$	2.11 $\times \;10^{4}$	6.75 $\times \;10^{3}$	1.50 $\times \;10^{9}$	1.32 $\times \;10^{5}$	1.68 $\times \;10^{4}$	5.96 $\times \;10^{6}$	$4.01\times 10^{3}$
		STD	2.97 $\times \;10^{4}$	6.52 $\times \;10^{3}$	2.96 $\times \;10^{3}$	6.28 $\times \;10^{8}$	4.14 $\times \;10^{5}$	2.04 $\times \;10^{4}$	1.70 $\times \;10^{7}$	3.88 $\times \;10^{3}$
	50	MFE	1.33 $\times \;10^{5}$	2.98 $\times \;10^{4}$	9.64 $\times \;10^{8}$	1.13 $\times \;10^{10}$	4.05 $\times \;10^{6}$	7.72 $\times \;10^{5}$	3.60 $\times \;10^{8}$	$9.00\times 10^{3}$
		STD	6.94 $\times \;10^{4}$	8.24 $\times \;10^{3}$	6.30 $\times \;10^{8}$	3.14 $\times \;10^{9}$	7.25 $\times \;10^{6}$	5.67 $\times \;10^{5}$	1.04 $\times \;10^{9}$	7.02 $\times \;10^{3}$
F16	10	MFE	1.41 $\times \;10^{2}$	3.45 $\times \;10^{2}$	1.02 $\times \;10^{2}$	6.92 $\times \;10^{2}$	9.31 $\times \;10^{1}$	3.89 $\times \;10^{0}$	1.61 $\times \;10^{2}$	$3.15\times 10^{0}$
		STD	1.28 $\times \;10^{2}$	1.46 $\times \;10^{2}$	8.49 $\times \;10^{1}$	1.84 $\times \;10^{2}$	9.15 $\times \;10^{1}$	1.62 $\times \;10^{0}$	1.30 $\times \;10^{2}$	4.50 $\times \;10^{0}$
	30	MFE	1.34 $\times \;10^{3}$	2.62 $\times \;10^{3}$	8.04 $\times \;10^{2}$	4.37 $\times \;10^{3}$	7.68 $\times \;10^{2}$	$2.62\times 10^{2}$	1.53 $\times \;10^{3}$	4.10 $\times \;10^{2}$
		STD	3.48 $\times \;10^{2}$	6.36 $\times \;10^{2}$	1.87 $\times \;10^{2}$	3.80 $\times \;10^{2}$	2.40 $\times \;10^{2}$	2.95 $\times \;10^{2}$	2.55 $\times \;10^{2}$	2.85 $\times \;10^{2}$
	50	MFE	1.89 $\times \;10^{3}$	4.53 $\times \;10^{3}$	1.66 $\times \;10^{3}$	7.68 $\times \;10^{3}$	1.23 $\times \;10^{3}$	$6.62\times 10^{2}$	2.33 $\times \;10^{3}$	1.03 $\times \;10^{3}$
		STD	6.41 $\times \;10^{2}$	1.03 $\times \;10^{3}$	3.10 $\times \;10^{2}$	6.51 $\times \;10^{2}$	2.67 $\times \;10^{2}$	3.20 $\times \;10^{2}$	4.67 $\times \;10^{2}$	3.82 $\times \;10^{2}$
F17	10	MFE	6.33 $\times \;10^{1}$	2.09 $\times \;10^{2}$	2.72 $\times \;10^{1}$	3.58 $\times \;10^{2}$	4.49 $\times \;10^{1}$	3.69 $\times \;10^{1}$	7.72 $\times \;10^{1}$	$6.92\times 10^{0}$
		STD	2.90 $\times \;10^{1}$	1.20 $\times \;10^{2}$	1.39 $\times \;10^{1}$	9.94 $\times \;10^{1}$	1.16 $\times \;10^{1}$	5.56 $\times \;10^{0}$	1.03 $\times \;10^{1}$	7.65 $\times \;10^{0}$
	30	MFE	4.75 $\times \;10^{2}$	9.51 $\times \;10^{2}$	3.62 $\times \;10^{2}$	2.34 $\times \;10^{3}$	2.58 $\times \;10^{2}$	1.16 $\times \;10^{2}$	5.78 $\times \;10^{2}$	$1.07\times 10^{2}$
		STD	2.46 $\times \;10^{2}$	3.28 $\times \;10^{2}$	1.62 $\times \;10^{2}$	3.71 $\times \;10^{2}$	1.35 $\times \;10^{2}$	7.79 $\times \;10^{1}$	2.21 $\times \;10^{2}$	6.14 $\times \;10^{1}$
	50	MFE	1.78 $\times \;10^{3}$	2.33 $\times \;10^{3}$	1.38 $\times \;10^{3}$	3.64 $\times \;10^{4}$	9.96 $\times \;10^{2}$	8.08 $\times \;10^{2}$	1.82 $\times \;10^{3}$	$6.85\times 10^{2}$
		STD	4.02 $\times \;10^{2}$	4.04 $\times \;10^{2}$	3.00 $\times \;10^{2}$	2.42 $\times \;10^{4}$	2.43 $\times \;10^{2}$	3.90 $\times \;10^{2}$	3.42 $\times \;10^{2}$	2.92 $\times \;10^{2}$
F18	10	MFE	2.27 $\times \;10^{4}$	1.44 $\times \;10^{4}$	1.03 $\times \;10^{3}$	3.68 $\times \;10^{7}$	2.20 $\times \;10^{4}$	3.84 $\times \;10^{3}$	4.57 $\times \;10^{4}$	$2.36\times 10^{3}$
		STD	1.49 $\times \;10^{4}$	9.58 $\times \;10^{3}$	2.45 $\times \;10^{3}$	2.59 $\times \;10^{7}$	1.14 $\times \;10^{4}$	3.06 $\times \;10^{3}$	1.56 $\times \;10^{4}$	1.69 $\times \;10^{3}$
	30	MFE	1.30 $\times \;10^{6}$	1.39 $\times \;10^{6}$	5.61 $\times \;10^{5}$	9.09 $\times \;10^{7}$	9.08 $\times \;10^{5}$	1.64 $\times \;10^{5}$	4.15 $\times \;10^{6}$	$8.99\times 10^{4}$
		STD	1.20 $\times \;10^{6}$	2.28 $\times \;10^{6}$	5.18 $\times \;10^{5}$	4.59 $\times \;10^{7}$	1.68 $\times \;10^{6}$	1.25 $\times \;10^{5}$	8.06 $\times \;10^{6}$	7.09 $\times \;10^{4}$
	50	MFE	2.93 $\times \;10^{6}$	1.79 $\times \;10^{7}$	1.17 $\times \;10^{7}$	2.13 $\times \;10^{8}$	1.74 $\times \;10^{6}$	9.07 $\times \;10^{5}$	1.19 $\times \;10^{7}$	$3.02\times 10^{5}$
		STD	1.95 $\times \;10^{6}$	2.09 $\times \;10^{7}$	6.80 $\times \;10^{6}$	7.97 $\times \;10^{7}$	1.53 $\times \;10^{6}$	3.01 $\times \;10^{5}$	1.35 $\times \;10^{7}$	1.53 $\times \;10^{5}$
F19	10	MFE	2.71 $\times \;10^{3}$	2.31 $\times \;10^{4}$	2.23 $\times \;10^{2}$	1.20 $\times \;10^{6}$	4.73 $\times \;10^{3}$	2.51 $\times \;10^{1}$	5.37 $\times \;10^{3}$	$3.15\times 10^{0}$
		STD	2.76 $\times \;10^{3}$	2.21 $\times \;10^{4}$	5.65 $\times \;10^{2}$	2.08 $\times \;10^{6}$	5.89 $\times \;10^{3}$	1.16 $\times \;10^{1}$	5.32 $\times \;10^{3}$	2.08 $\times \;10^{0}$
	30	MFE	3.88 $\times \;10^{5}$	1.06 $\times \;10^{6}$	6.37 $\times \;10^{4}$	2.09 $\times \;10^{9}$	6.05 $\times \;10^{5}$	7.25 $\times \;10^{3}$	2.86 $\times \;10^{6}$	$6.63\times 10^{3}$
		STD	2.71 $\times \;10^{5}$	1.11 $\times \;10^{5}$	6.48 $\times \;10^{4}$	7.04 $\times \;10^{8}$	1.20 $\times \;10^{6}$	7.52 $\times \;10^{3}$	3.42 $\times \;10^{6}$	4.77 $\times \;10^{3}$
	50	MFE	6.60 $\times \;10^{5}$	4.61 $\times \;10^{5}$	1.90 $\times \;10^{8}$	4.18 $\times \;10^{9}$	7.94 $\times \;10^{5}$	5.37 $\times \;10^{5}$	1.96 $\times \;10^{7}$	$1.97\times 10^{4}$
		STD	4.36 $\times \;10^{5}$	1.24 $\times \;10^{4}$	2.06 $\times \;10^{8}$	4.86 $\times \;10^{8}$	5.58 $\times \;10^{5}$	7.04 $\times \;10^{5}$	5.89 $\times \;10^{7}$	1.23 $\times \;10^{4}$
F20	10	MFE	8.59 $\times \;10^{1}$	1.25 $\times \;10^{2}$	$1.16\times 10^{0}$	2.88 $\times \;10^{2}$	5.49 $\times \;10^{1}$	2.66 $\times \;10^{1}$	9.54 $\times \;10^{1}$	2.70 $\times \;10^{0}$
		STD	3.89 $\times \;10^{1}$	5.37 $\times \;10^{1}$	2.82 $\times \;10^{0}$	8.06 $\times \;10^{1}$	5.20 $\times \;10^{1}$	4.50 $\times \;10^{0}$	3.94 $\times \;10^{1}$	6.16 $\times \;10^{0}$
	30	MFE	4.25 $\times \;10^{2}$	6.60 $\times \;10^{2}$	3.08 $\times \;10^{2}$	1.29 $\times \;10^{3}$	3.19 $\times \;10^{2}$	1.52 $\times \;10^{2}$	6.18 $\times \;10^{2}$	$1.13\times 10^{2}$
		STD	1.36 $\times \;10^{2}$	1.71 $\times \;10^{2}$	6.46 $\times \;10^{1}$	1.72 $\times \;10^{2}$	1.15 $\times \;10^{2}$	1.15 $\times \;10^{2}$	1.44 $\times \;10^{2}$	7.62 $\times \;10^{1}$
	50	MFE	9.88 $\times \;10^{2}$	1.43 $\times \;10^{3}$	8.13 $\times \;10^{2}$	2.73 $\times \;10^{3}$	7.57 $\times \;10^{2}$	6.07 $\times \;10^{2}$	1.15 $\times \;10^{3}$	$4.57\times 10^{2}$
		STD	2.97 $\times \;10^{2}$	2.55 $\times \;10^{2}$	1.87 $\times \;10^{2}$	1.67 $\times \;10^{2}$	1.72 $\times \;10^{2}$	3.14 $\times \;10^{2}$	2.54 $\times \;10^{2}$	3.88 $\times \;10^{2}$
Rank	10	w/t/l	0/0/10	0/0/10	1/0/9	0/0/10	0/0/10	1/0/9	0/0/10	8/0/2
	30	w/t/l	0/0/10	0/0/10	0/0/10	0/0/10	0/0/10	1/0/9	0/0/10	9/0/1
	50	w/t/l	0/0/10	0/0/10	0/0/10	0/0/10	0/0/10	1/0/9	0/0/10	9/0/1

**Bold font** denotes the best result.

**Table 5:** The comparison of obtained solutions for composition functions of IEEE CEC 2017.

F	D	Index	Algorithm							
			AO (2025)	AOA (2021)	CBOA (2022)	NOA (2023)	GWO (2024)	IGWO (2021)	AGWO (2022)	HSB-GWO
F21	10	MFE	$1.46\times 10^{2}$	2.08 $\times \;10^{2}$	1.69 $\times \;10^{2}$	2.61 $\times \;10^{2}$	2.03 $\times \;10^{2}$	1.57 $\times \;10^{2}$	1.60 $\times \;10^{2}$	1.85 $\times \;10^{2}$
		STD	5.57 $\times \;10^{1}$	3.23 $\times \;10^{1}$	5.30 $\times \;10^{1}$	5.83 $\times \;10^{1}$	3.35 $\times \;10^{1}$	5.84 $\times \;10^{1}$	5.43 $\times \;10^{1}$	4.38 $\times \;10^{1}$
	30	MFE	3.28 $\times \;10^{2}$	5.01 $\times \;10^{2}$	3.22 $\times \;10^{2}$	6.80 $\times \;10^{2}$	2.80 $\times \;10^{2}$	2.55 $\times \;10^{2}$	3.72 $\times \;10^{2}$	$2.35\times 10^{2}$
		STD	2.66 $\times \;10^{1}$	5.41 $\times \;10^{1}$	1.93 $\times \;10^{1}$	3.80 $\times \;10^{1}$	3.79 $\times \;10^{1}$	4.10 $\times \;10^{1}$	3.61 $\times \;10^{1}$	9.35 $\times \;10^{0}$
	50	MFE	5.06 $\times \;10^{2}$	9.08 $\times \;10^{2}$	4.88 $\times \;10^{2}$	1.18 $\times \;10^{3}$	3.63 $\times \;10^{2}$	4.29 $\times \;10^{2}$	5.98 $\times \;10^{2}$	$2.82\times 10^{2}$
		STD	4.30 $\times \;10^{1}$	6.40 $\times \;10^{1}$	4.15 $\times \;10^{1}$	6.17 $\times \;10^{1}$	2.85 $\times \;10^{1}$	4.14 $\times \;10^{1}$	5.55 $\times \;10^{1}$	1.62 $\times \;10^{1}$
F22	10	MFE	1.07 $\times \;10^{2}$	3.51 $\times \;10^{2}$	9.75 $\times \;10^{1}$	9.12 $\times \;10^{2}$	1.06 $\times \;10^{2}$	$1.00\times 10^{2}$	1.84 $\times \;10^{2}$	$1.00\times 10^{2}$
		STD	1.75 $\times \;10^{0}$	9.76 $\times \;10^{1}$	2.32 $\times \;10^{1}$	2.11 $\times \;10^{2}$	4.29 $\times \;10^{0}$	2.35 $\times \;10^{1}$	3.72 $\times \;10^{1}$	4.55 $\times \;10^{-1}$
	30	MFE	3.18 $\times \;10^{2}$	5.52 $\times \;10^{3}$	1.60 $\times \;10^{3}$	8.01 $\times \;10^{3}$	1.72 $\times \;10^{3}$	1.02 $\times \;10^{2}$	1.40 $\times \;10^{3}$	$1.00\times 10^{2}$
		STD	9.08 $\times \;10^{2}$	9.71 $\times \;10^{2}$	6.22 $\times \;10^{2}$	6.65 $\times \;10^{2}$	1.34 $\times \;10^{3}$	5.96 $\times \;10^{-1}$	8.56 $\times \;10^{2}$	2.56 $\times \;10^{-13}$
	50	MFE	6.68 $\times \;10^{3}$	1.29 $\times \;10^{4}$	6.18 $\times \;10^{3}$	1.55 $\times \;10^{4}$	5.80 $\times \;10^{3}$	1.03 $\times \;10^{4}$	9.47 $\times \;10^{3}$	$3.94\times 10^{3}$
		STD	1.67 $\times \;10^{3}$	6.63 $\times \;10^{2}$	8.84 $\times \;10^{2}$	4.71 $\times \;10^{2}$	1.33 $\times \;10^{3}$	2.39 $\times \;10^{3}$	1.41 $\times \;10^{3}$	2.29 $\times \;10^{3}$
F23	10	MFE	3.37 $\times \;10^{2}$	3.95 $\times \;10^{2}$	3.15 $\times \;10^{2}$	4.56 $\times \;10^{2}$	3.17 $\times \;10^{2}$	3.11 $\times \;10^{2}$	3.65 $\times \;10^{2}$	$3.09\times 10^{2}$
		STD	1.09 $\times \;10^{1}$	2.76 $\times \;10^{1}$	7.29 $\times \;10^{0}$	2.69 $\times \;10^{1}$	6.25 $\times \;10^{0}$	4.94 $\times \;10^{0}$	7.94 $\times \;10^{0}$	3.23 $\times \;10^{0}$
	30	MFE	5.59 $\times \;10^{2}$	1.03 $\times \;10^{3}$	4.85 $\times \;10^{2}$	1.21 $\times \;10^{3}$	4.62 $\times \;10^{2}$	3.95 $\times \;10^{2}$	6.33 $\times \;10^{2}$	$3.96\times 10^{2}$
		STD	5.41 $\times \;10^{1}$	7.99 $\times \;10^{1}$	4.09 $\times \;10^{1}$	7.46 $\times \;10^{1}$	4.87 $\times \;10^{1}$	3.39 $\times \;10^{1}$	7.28 $\times \;10^{1}$	1.62 $\times \;10^{1}$
	50	MFE	9.56 $\times \;10^{2}$	1.98 $\times \;10^{3}$	8.55 $\times \;10^{2}$	2.11 $\times \;10^{3}$	6.09 $\times \;10^{2}$	7.34 $\times \;10^{2}$	9.90 $\times \;10^{2}$	$5.04\times 10^{2}$
		STD	7.65 $\times \;10^{1}$	2.00 $\times \;10^{2}$	7.45 $\times \;10^{1}$	7.64 $\times \;10^{1}$	3.40 $\times \;10^{1}$	1.64 $\times \;10^{1}$	1.11 $\times \;10^{2}$	2.41 $\times \;10^{1}$
F24	10	MFE	3.51 $\times \;10^{2}$	3.95 $\times \;10^{2}$	2.48 $\times \;10^{2}$	4.39 $\times \;10^{2}$	3.42 $\times \;10^{2}$	3.12 $\times \;10^{2}$	3.07 $\times \;10^{2}$	$3.14\times 10^{2}$
		STD	6.01 $\times \;10^{1}$	5.99 $\times \;10^{1}$	1.25 $\times \;10^{2}$	3.02 $\times \;10^{1}$	1.34 $\times \;10^{1}$	7.27 $\times \;10^{1}$	8.56 $\times \;10^{1}$	7.35 $\times \;10^{1}$
	30	MFE	6.15 $\times \;10^{2}$	1.28 $\times \;10^{3}$	7.82 $\times \;10^{2}$	1.37 $\times \;10^{3}$	5.09 $\times \;10^{2}$	4.63 $\times \;10^{2}$	6.93 $\times \;10^{2}$	$4.62\times 10^{2}$
		STD	4.75 $\times \;10^{1}$	1.52 $\times \;10^{2}$	7.12 $\times \;10^{1}$	1.17 $\times \;10^{2}$	5.12 $\times \;10^{1}$	2.93 $\times \;10^{1}$	9.27 $\times \;10^{1}$	1.52 $\times \;10^{1}$
	50	MFE	9.17 $\times \;10^{2}$	2.25 $\times \;10^{3}$	1.40 $\times \;10^{3}$	2.46 $\times \;10^{3}$	6.60 $\times \;10^{2}$	8.19 $\times \;10^{2}$	1.06 $\times \;10^{3}$	$5.85\times 10^{2}$
		STD	6.15 $\times \;10^{1}$	2.24 $\times \;10^{2}$	1.39 $\times \;10^{2}$	1.28 $\times \;10^{2}$	6.61 $\times \;10^{1}$	2.59 $\times \;10^{1}$	1.02 $\times \;10^{2}$	2.13 $\times \;10^{1}$
F25	10	MFE	4.23 $\times \;10^{2}$	5.09 $\times \;10^{2}$	4.38 $\times \;10^{2}$	9.93 $\times \;10^{2}$	4.29 $\times \;10^{2}$	$3.98\times 10^{2}$	4.55 $\times \;10^{2}$	4.10 $\times \;10^{2}$
		STD	2.46 $\times \;10^{1}$	9.42 $\times \;10^{1}$	1.99 $\times \;10^{1}$	1.94 $\times \;10^{2}$	2.02 $\times \;10^{1}$	3.62 $\times \;10^{-1}$	2.83 $\times \;10^{1}$	2.05 $\times \;10^{1}$
	30	MFE	4.08 $\times \;10^{2}$	1.98 $\times \;10^{3}$	6.58 $\times \;10^{2}$	8.25 $\times \;10^{3}$	4.54 $\times \;10^{2}$	3.87 $\times \;10^{2}$	6.16 $\times \;10^{2}$	$3.86\times 10^{2}$
		STD	1.85 $\times \;10^{1}$	5.60 $\times \;10^{2}$	5.17 $\times \;10^{1}$	1.76 $\times \;10^{3}$	2.22 $\times \;10^{1}$	1.94 $\times \;10^{-1}$	1.24 $\times \;10^{2}$	1.25 $\times \;10^{0}$
	50	MFE	6.00 $\times \;10^{2}$	1.09 $\times \;10^{4}$	4.74 $\times \;10^{3}$	3.31 $\times \;10^{4}$	9.24 $\times \;10^{2}$	5.43 $\times \;10^{2}$	1.90 $\times \;10^{3}$	$5.41\times 10^{2}$
		STD	3.07 $\times \;10^{1}$	1.74 $\times \;10^{3}$	5.40 $\times \;10^{2}$	3.79 $\times \;10^{3}$	1.76 $\times \;10^{2}$	2.54 $\times \;10^{1}$	7.91 $\times \;10^{2}$	3.63 $\times \;10^{1}$
F26	10	MFE	3.93 $\times \;10^{2}$	9.13 $\times \;10^{2}$	3.80 $\times \;10^{2}$	1.38 $\times \;10^{3}$	3.57 $\times \;10^{2}$	$3.00\times 10^{2}$	6.71 $\times \;10^{2}$	$3.00\times 10^{2}$
		STD	1.49 $\times \;10^{2}$	2.76 $\times \;10^{2}$	1.08 $\times \;10^{2}$	2.26 $\times \;10^{2}$	2.03 $\times \;10^{2}$	5.45 $\times \;10^{-2}$	2.08 $\times \;10^{2}$	1.48 $\times \;10^{-13}$
	30	MFE	1.94 $\times \;10^{3}$	6.95 $\times \;10^{3}$	4.14 $\times \;10^{3}$	9.83 $\times \;10^{3}$	1.99 $\times \;10^{3}$	1.23 $\times \;10^{3}$	3.88 $\times \;10^{3}$	$9.79\times 10^{2}$
		STD	1.66 $\times \;10^{3}$	8.70 $\times \;10^{2}$	6.68 $\times \;10^{2}$	7.51 $\times \;10^{2}$	3.18 $\times \;10^{2}$	2.78 $\times \;10^{2}$	1.14 $\times \;10^{3}$	5.01 $\times \;10^{2}$
	50	MFE	1.74 $\times \;10^{3}$	1.32 $\times \;10^{4}$	9.27 $\times \;10^{3}$	1.96 $\times \;10^{4}$	3.12 $\times \;10^{3}$	4.14 $\times \;10^{3}$	8.51 $\times \;10^{3}$	$1.39\times 10^{3}$
		STD	2.45 $\times \;10^{3}$	1.09 $\times \;10^{3}$	7.73 $\times \;10^{2}$	1.51 $\times \;10^{3}$	4.32 $\times \;10^{2}$	2.29 $\times \;10^{2}$	1.32 $\times \;10^{3}$	9.55 $\times \;10^{2}$
F27	10	MFE	3.99 $\times \;10^{2}$	4.93 $\times \;10^{2}$	4.03 $\times \;10^{2}$	5.33 $\times \;10^{2}$	3.94 $\times \;10^{2}$	$3.90\times 10^{2}$	4.21 $\times \;10^{2}$	$3.90\times 10^{2}$
		STD	3.13 $\times \;10^{0}$	3.83 $\times \;10^{1}$	1.54 $\times \;10^{1}$	2.92 $\times \;10^{1}$	8.00 $\times \;10^{0}$	3.90 $\times \;10^{-1}$	1.99 $\times \;10^{1}$	8.02 $\times \;10^{-1}$
	30	MFE	5.80 $\times \;10^{2}$	1.59 $\times \;10^{3}$	6.05 $\times \;10^{2}$	1.60 $\times \;10^{3}$	5.37 $\times \;10^{2}$	$4.94\times 10^{2}$	6.20 $\times \;10^{2}$	5.00 $\times \;10^{2}$
		STD	2.72 $\times \;10^{1}$	3.11 $\times \;10^{2}$	4.54 $\times \;10^{1}$	1.83 $\times \;10^{2}$	1.66 $\times \;10^{1}$	9.73 $\times \;10^{0}$	4.43 $\times \;10^{1}$	9.84 $\times \;10^{0}$
	50	MFE	9.72 $\times \;10^{2}$	3.51 $\times \;10^{3}$	1.20 $\times \;10^{3}$	3.88 $\times \;10^{3}$	8.01 $\times \;10^{2}$	6.03 $\times \;10^{2}$	1.24 $\times \;10^{3}$	$5.62\times 10^{2}$
		STD	1.65 $\times \;10^{2}$	4.42 $\times \;10^{2}$	1.83 $\times \;10^{2}$	3.44 $\times \;10^{2}$	7.47 $\times \;10^{1}$	4.72 $\times \;10^{1}$	2.47 $\times \;10^{2}$	4.94 $\times \;10^{1}$
F28	10	MFE	4.98 $\times \;10^{2}$	7.34 $\times \;10^{2}$	5.40 $\times \;10^{2}$	9.60 $\times \;10^{2}$	5.41 $\times \;10^{2}$	$3.78\times 10^{2}$	5.70 $\times \;10^{2}$	4.69 $\times \;10^{2}$
		STD	1.10 $\times \;10^{2}$	1.52 $\times \;10^{2}$	1.43 $\times \;10^{2}$	1.60 $\times \;10^{2}$	9.69 $\times \;10^{1}$	1.38 $\times \;10^{2}$	1.25 $\times \;10^{2}$	1.57 $\times \;10^{2}$
	30	MFE	4.72 $\times \;10^{2}$	3.24 $\times \;10^{3}$	1.46 $\times \;10^{3}$	6.18 $\times \;10^{3}$	5.46 $\times \;10^{2}$	4.14 $\times \;10^{2}$	8.98 $\times \;10^{2}$	$3.27\times 10^{2}$
		STD	1.95 $\times \;10^{1}$	6.50 $\times \;10^{2}$	1.96 $\times \;10^{2}$	8.61 $\times \;10^{2}$	6.91 $\times \;10^{1}$	9.68 $\times \;10^{0}$	2.50 $\times \;10^{2}$	4.19 $\times \;10^{1}$
	50	MFE	5.52 $\times \;10^{2}$	8.18 $\times \;10^{3}$	4.70 $\times \;10^{3}$	1.40 $\times \;10^{4}$	1.18 $\times \;10^{3}$	$4.67\times 10^{2}$	2.20 $\times \;10^{3}$	4.78 $\times \;10^{2}$
		STD	3.24 $\times \;10^{1}$	1.21 $\times \;10^{3}$	5.71 $\times \;10^{2}$	1.20 $\times \;10^{3}$	3.12 $\times \;10^{2}$	8.31 $\times \;10^{0}$	5.75 $\times \;10^{2}$	2.25 $\times \;10^{1}$
F29	10	MFE	3.21 $\times \;10^{2}$	4.14 $\times \;10^{2}$	2.67 $\times \;10^{2}$	6.88 $\times \;10^{2}$	2.72 $\times \;10^{2}$	2.53 $\times \;10^{2}$	3.49 $\times \;10^{2}$	$2.37\times 10^{2}$
		STD	4.50 $\times \;10^{1}$	8.86 $\times \;10^{1}$	1.60 $\times \;10^{1}$	1.06 $\times \;10^{2}$	3.29 $\times \;10^{1}$	1.34 $\times \;10^{1}$	3.73 $\times \;10^{1}$	6.96 $\times \;10^{0}$
	30	MFE	1.29 $\times \;10^{3}$	2.92 $\times \;10^{3}$	1.06 $\times \;10^{3}$	4.61 $\times \;10^{3}$	7.59 $\times \;10^{2}$	5.24 $\times \;10^{2}$	1.63 $\times \;10^{3}$	$4.88\times 10^{2}$
		STD	2.24 $\times \;10^{2}$	5.93 $\times \;10^{2}$	1.94 $\times \;10^{2}$	7.71 $\times \;10^{2}$	1.67 $\times \;10^{2}$	9.39 $\times \;10^{1}$	2.50 $\times \;10^{2}$	7.36 $\times \;10^{1}$
	50	MFE	2.18 $\times \;10^{3}$	1.06 $\times \;10^{4}$	4.03 $\times \;10^{3}$	3.85 $\times \;10^{4}$	1.34 $\times \;10^{3}$	9.73 $\times \;10^{2}$	3.33 $\times \;10^{3}$	$5.10\times 10^{2}$
		STD	4.54 $\times \;10^{2}$	3.17 $\times \;10^{3}$	1.12 $\times \;10^{3}$	2.23 $\times \;10^{4}$	2.89 $\times \;10^{2}$	1.17 $\times \;10^{2}$	6.98 $\times \;10^{2}$	1.76 $\times \;10^{2}$
F30	10	MFE	3.10 $\times \;10^{5}$	7.62 $\times \;10^{6}$	1.48 $\times \;10^{5}$	1.84 $\times \;10^{7}$	2.72 $\times \;10^{5}$	1.15 $\times \;10^{5}$	6.79 $\times \;10^{5}$	$2.70\times 10^{5}$
		STD	5.55 $\times \;10^{5}$	8.31 $\times \;10^{6}$	7.51 $\times \;10^{4}$	7.16 $\times \;10^{6}$	4.58 $\times \;10^{5}$	2.81 $\times \;10^{5}$	8.60 $\times \;10^{5}$	4.90 $\times \;10^{5}$
	30	MFE	2.34 $\times \;10^{6}$	1.74 $\times \;10^{7}$	9.97 $\times \;10^{6}$	1.06 $\times \;10^{9}$	3.24 $\times \;10^{6}$	6.68 $\times \;10^{4}$	1.43 $\times \;10^{7}$	$7.40\times 10^{3}$
		STD	1.16 $\times \;10^{6}$	1.32 $\times \;10^{7}$	1.32 $\times \;10^{7}$	4.47 $\times \;10^{8}$	1.58 $\times \;10^{6}$	4.77 $\times \;10^{4}$	1.10 $\times \;10^{7}$	4.46 $\times \;10^{3}$
	50	MFE	2.59 $\times \;10^{7}$	5.17 $\times \;10^{8}$	6.98 $\times \;10^{8}$	7.07 $\times \;10^{9}$	8.05 $\times \;10^{7}$	1.76 $\times \;10^{7}$	1.57 $\times \;10^{8}$	$8.73\times 10^{5}$
		STD	6.12 $\times \;10^{6}$	9.45 $\times \;10^{8}$	5.56 $\times \;10^{8}$	1.84 $\times \;10^{9}$	2.47 $\times \;10^{7}$	6.24 $\times \;10^{6}$	6.74 $\times \;10^{7}$	2.06 $\times \;10^{5}$
Rank	10	w/t/l	1/0/9	0/0/10	0/0/10	0/0/10	0/0/10	2/3/5	1/0/9	4/3/3
	30	w/t/l	0/0/10	0/0/10	0/0/10	0/0/10	0/0/10	1/0/9	0/0/10	9/0/1
	50	w/t/l	0/0/10	0/0/10	0/0/10	0/0/10	0/0/10	1/0/9	0/0/10	9/0/1

**Bold font** denotes the best result.

**Table 6:** Overall effectiveness of the proposed HSB-GWO and other competitor algorithms for IEEE CEC 2017.

	AO (w/t/l)	AOA (w/t/l)	CBOA (w/t/l)	NOA (w/t/l)	GWO (w/t/l)	IGWO (w/t/l)	AGWO (w/t/l)	HSB-GWO (w/t/l)
D=10	1/0/28	0/0/29	3/0/26	0/0/29	0/0/29	3/3/23	1/0/28	19/3/7
D=30	0/0/29	0/0/29	1/0/28	0/0/29	0/0/29	4/0/25	0/0/29	25/0/4
D=50	0/0/29	0/0/29	1/0/28	0/0/29	0/0/29	2/0/27	0/0/29	25/0/4
Total	1/0/86	0/0/87	5/0/82	0/0/87	0/0/87	9/3/75	1/0/86	69/3/15
OE	1.15%	0%	5.75%	0%	0%	13.79%	1.15%	**82.76%**

**Bold font** denotes the best result.

**Table 7:** The comparison of obtained solutions for IEEE CEC 2019.

F	Index	Algorithm							
		AO	AOA	CBOA	NOA	GWO	IGWO	AGWO	HSB-GWO
F1	MFE	2.18 $\times \;10^{-12}$	6.44 $\times \;10^{-8}$	$0.00\times 10^{0}$	4.20 $\times \;10^{8}$	1.21 $\times \;10^{4}$	3.85 $\times \;10^{3}$	1.56 $\times \;10^{-9}$	$0.00\times 10^{0}$
	STD	9.72 $\times \;10^{-12}$	2.88 $\times \;10^{-7}$	0.00 $\times \;10^{0}$	2.10 $\times \;10^{8}$	4.76 $\times \;10^{4}$	6.71 $\times \;10^{3}$	6.44 $\times \;10^{-9}$	0.00 $\times \;10^{0}$
F2	MFE	3.97 $\times \;10^{0}$	5.87 $\times \;10^{3}$	3.82 $\times \;10^{0}$	1.63 $\times \;10^{4}$	2.64 $\times \;10^{2}$	4.94 $\times \;10^{2}$	$3.36\times 10^{0}$	3.54 $\times \;10^{0}$
	STD	1.36 $\times \;10^{-1}$	1.69 $\times \;10^{3}$	1.48 $\times \;10^{-1}$	3.88 $\times \;10^{3}$	2.22 $\times \;10^{2}$	1.33 $\times \;10^{2}$	2.28 $\times \;10^{-1}$	3.87 $\times \;10^{-1}$
F3	MFE	2.64 $\times \;10^{0}$	7.54 $\times \;10^{0}$	$2.09\times 10^{-1}$	1.08 $\times \;10^{1}$	9.93 $\times \;10^{-1}$	9.40 $\times \;10^{-1}$	4.59 $\times \;10^{0}$	4.47 $\times \;10^{-1}$
	STD	1.01 $\times \;10^{0}$	1.26 $\times \;10^{0}$	2.06 $\times \;10^{-1}$	3.74 $\times \;10^{-1}$	1.04 $\times \;10^{0}$	7.47 $\times \;10^{-1}$	1.41 $\times \;10^{0}$	7.68 $\times \;10^{-1}$
F4	MFE	2.73 $\times \;10^{1}$	4.08 $\times \;10^{1}$	1.09 $\times \;10^{1}$	1.08 $\times \;10^{2}$	8.67 $\times \;10^{0}$	1.54 $\times \;10^{1}$	4.45 $\times \;10^{1}$	$6.15\times 10^{0}$
	STD	1.00 $\times \;10^{1}$	1.29 $\times \;10^{1}$	2.58 $\times \;10^{0}$	1.59 $\times \;10^{1}$	4.12 $\times \;10^{0}$	4.77 $\times \;10^{0}$	8.69 $\times \;10^{0}$	2.70 $\times \;10^{0}$
F5	MFE	6.33 $\times \;10^{-1}$	2.66 $\times \;10^{1}$	2.76 $\times \;10^{-1}$	8.84 $\times \;10^{1}$	6.17 $\times \;10^{-1}$	4.88 $\times \;10^{-1}$	7.77 $\times \;10^{0}$	$1.50\times 10^{-1}$
	STD	1.84 $\times \;10^{-1}$	1.37 $\times \;10^{1}$	2.27 $\times \;10^{-1}$	2.22 $\times \;10^{1}$	5.95 $\times \;10^{-1}$	9.14 $\times \;10^{-2}$	9.63 $\times \;10^{0}$	1.14 $\times \;10^{-1}$
F6	MFE	2.63 $\times \;10^{0}$	8.91 $\times \;10^{0}$	4.37 $\times \;10^{-1}$	1.18 $\times \;10^{1}$	7.46 $\times \;10^{-1}$	4.07 $\times \;10^{-1}$	5.68 $\times \;10^{0}$	$1.25\times 10^{-1}$
	STD	7.38 $\times \;10^{-1}$	1.38 $\times \;10^{0}$	5.07 $\times \;10^{-1}$	9.05 $\times \;10^{-1}$	6.03 $\times \;10^{-1}$	2.48 $\times \;10^{-1}$	1.10 $\times \;10^{0}$	3.70 $\times \;10^{-1}$
F7	MFE	8.81 $\times \;10^{2}$	1.10 $\times \;10^{3}$	2.68 $\times \;10^{2}$	2.18 $\times \;10^{3}$	5.59 $\times \;10^{2}$	5.49 $\times \;10^{2}$	1.35 $\times \;10^{3}$	$1.52\times 10^{2}$
	STD	2.74 $\times \;10^{2}$	2.30 $\times \;10^{2}$	7.23 $\times \;10^{1}$	2.64 $\times \;10^{2}$	1.79 $\times \;10^{2}$	3.09 $\times \;10^{2}$	2.51 $\times \;10^{2}$	1.54 $\times \;10^{2}$
F8	MFE	3.02 $\times \;10^{0}$	3.58 $\times \;10^{0}$	1.69 $\times \;10^{0}$	4.17 $\times \;10^{0}$	2.31 $\times \;10^{0}$	1.58 $\times \;10^{0}$	3.26 $\times \;10^{0}$	$1.34\times 10^{0}$
	STD	2.96 $\times \;10^{-1}$	3.32 $\times \;10^{-1}$	3.91 $\times \;10^{-1}$	1.29 $\times \;10^{-1}$	4.40 $\times \;10^{-1}$	3.55 $\times \;10^{-1}$	2.09 $\times \;10^{-1}$	5.96 $\times \;10^{-1}$
F9	MFE	3.21 $\times \;10^{-1}$	2.95 $\times \;10^{-1}$	1.54 $\times \;10^{-1}$	3.32 $\times \;10^{0}$	1.33 $\times \;10^{-1}$	1.26 $\times \;10^{-1}$	4.73 $\times \;10^{-1}$	$7.30\times 10^{-2}$
	STD	9.80 $\times \;10^{-2}$	1.12 $\times \;10^{-1}$	4.53 $\times \;10^{-2}$	5.18 $\times \;10^{-1}$	5.74 $\times \;10^{-2}$	2.93 $\times \;10^{-2}$	6.72 $\times \;10^{-2}$	2.23 $\times \;10^{-2}$
F10	MFE	1.64 $\times \;10^{1}$	2.01 $\times \;10^{1}$	1.24 $\times \;10^{1}$	2.07 $\times \;10^{1}$	2.04 $\times \;10^{1}$	1.81 $\times \;10^{1}$	2.03 $\times \;10^{1}$	$8.02\times 10^{0}$
	STD	7.88 $\times \;10^{0}$	3.91 $\times \;10^{-2}$	7.43 $\times \;10^{0}$	9.47 $\times \;10^{-2}$	6.10 $\times \;10^{-2}$	6.23 $\times \;10^{0}$	7.45 $\times \;10^{-2}$	1.01 $\times \;10^{1}$
Rank	w/t/l	0/0/10	0/0/10	1/1/8	0/0/10	0/0/10	0/0/10	1/0/9	7/1/2

**Bold font** denotes the best result.

**Table 8:** Overall rank by Friedman test in IEEE CEC 2017.

**Alg.**	**D**	**F1**	**F3**	**F4**	**F5**	**F6**	**F7**	**F8**	**F9**	**F10**	**F11**	**F12**	**F13**	**F14**	**F15**	**F16**	**F17**
AO	10	4.85	3.00	3.75	4.65	5.00	4.95	5.15	4.95	4.50	5.60	5.70	5.70	5.00	5.20	4.85	5.00
	30	3.00	3.00	3.10	4.55	5.35	4.45	5.00	5.55	4.50	3.85	3.30	5.45	4.50	5.60	5.15	4.65
	50	3.00	3.30	2.75	4.45	5.25	4.15	4.90	5.00	3.85	2.75	2.65	2.25	4.65	3.60	4.55	5.20
AOA	10	6.65	6.60	6.60	6.30	6.80	6.70	6.40	6.85	5.95	5.45	4.50	4.65	6.85	6.95	6.70	6.80
	30	7.00	6.90	7.00	6.85	7.00	7.00	7.00	6.05	6.25	6.60	7.05	2.35	3.90	4.35	7.05	6.55
	50	7.00	6.90	7.00	7.00	7.00	6.90	7.00	6.50	6.45	6.95	7.05	5.95	4.10	2.10	6.95	6.55
CBOA	10	**1.00**	5.10	3.25	2.70	2.70	2.65	2.60	3.15	**2.00**	3.25	4.15	3.15	2.60	2.55	3.80	2.35
	30	5.90	5.80	5.95	4.15	3.70	4.70	3.90	4.05	**2.10**	5.45	5.95	4.60	5.95	2.10	3.25	4.55
	50	6.00	6.00	6.00	4.35	3.90	4.55	3.80	3.95	**1.70**	5.75	6.00	6.80	6.60	6.85	4.30	4.30
NOA	10	8.00	8.00	8.00	8.00	8.00	8.00	8.00	8.00	8.00	8.00	8.00	8.00	7.65	7.95	7.80	7.75
	30	8.00	8.00	8.00	8.00	8.00	8.00	8.00	8.00	8.00	8.00	7.95	8.00	8.00	8.00	7.95	8.00
	50	8.00	8.00	8.00	8.00	8.00	8.00	8.00	8.00	8.00	8.00	7.95	8.00	8.00	8.00	8.00	8.00
GWO	10	3.15	4.35	4.65	3.05	3.45	2.95	3.50	3.75	3.55	4.05	4.05	5.05	4.35	4.55	4.40	3.80
	30	4.05	4.10	3.65	3.25	3.30	2.70	3.00	3.00	3.15	3.25	3.75	3.55	4.15	5.00	3.20	3.20
	50	4.05	4.05	4.00	2.30	3.10	1.90	2.30	3.10	3.05	4.10	4.05	3.85	3.00	3.90	2.95	2.60
IGWO	10	3.70	2.00	2.60	2.85	2.85	2.80	2.55	2.40	3.70	1.80	1.85	1.95	2.80	2.65	2.10	3.25
	30	2.00	1.80	2.10	1.80	1.90	2.10	**1.55**	2.00	3.80	1.90	1.95	4.25	1.70	3.00	**1.45**	**1.85**
	50	**1.50**	1.80	2.00	2.75	2.00	2.80	3.40	2.00	5.75	2.25	2.45	3.25	2.75	4.75	**1.35**	2.40
AGWO	10	6.35	5.95	6.15	6.65	6.20	6.15	6.20	5.90	6.10	6.65	6.05	5.80	5.65	5.10	5.00	5.90
	30	5.05	5.20	5.00	6.00	5.65	5.85	5.80	6.35	5.60	5.85	5.00	5.90	6.15	6.25	5.75	5.25
	50	4.95	4.75	5.00	5.90	5.75	5.95	5.60	6.45	5.40	5.20	4.85	4.90	5.75	5.80	5.70	5.45
HSB-GWO	10	2.30	**1.00**	**1.00**	**1.80**	**1.00**	**1.80**	**1.60**	**1.00**	2.20	**1.20**	**1.70**	**1.70**	**1.10**	**1.05**	**1.35**	**1.15**
	30	**1.00**	**1.20**	**1.20**	**1.40**	**1.10**	**1.20**	1.75	**1.00**	2.60	**1.10**	**1.05**	**1.90**	**1.65**	**1.70**	2.20	1.95
	50	**1.50**	**1.20**	**1.25**	**1.25**	**1.00**	**1.75**	**1.00**	**1.00**	1.80	**1.00**	**1.00**	**1.00**	**1.15**	**1.00**	2.20	**1.50**
**Alg.**	**D**	**F18**	**F19**	**F20**	**F21**	**F22**	**F23**	**F24**	**F25**	**F26**	**F27**	**F28**	**F29**	**F30**	**Avg. Rank**	**Overall Rank**	
AO	10	5.20	5.00	5.70	3.45	4.30	4.90	5.40	3.80	4.05	4.45	3.75	5.20	3.90	4.69	5	
	30	5.25	4.60	4.70	4.35	3.15	5.05	4.25	2.75	2.95	4.40	3.05	4.70	3.50	4.17	4	
	50	4.25	3.75	4.70	4.60	3.55	5.30	4.15	2.80	1.80	4.20	2.90	4.05	2.85	3.90	4	
AOA	10	4.45	6.45	6.35	5.65	6.90	6.95	6.60	5.85	6.80	7.15	6.60	6.60	7.10	6.55	7	
	30	4.85	6.05	6.45	7.00	7.00	7.05	7.20	7.00	7.05	7.40	7.00	7.05	6.45	6.48	7	
	50	5.95	3.55	6.55	7.00	6.85	7.25	7.20	7.00	7.00	7.20	7.00	7.05	6.30	6.48	7	
CBOA	10	**1.35**	2.70	**1.30**	4.10	2.15	2.85	2.90	5.20	4.05	4.40	4.25	3.35	4.00	2.79	3	
	30	3.90	3.30	3.45	4.30	5.05	3.55	5.65	5.80	5.50	5.15	5.95	4.20	5.45	4.59	5	
	50	5.90	6.95	3.45	4.20	2.85	4.20	5.95	6.00	5.75	5.40	5.95	5.65	6.45	5.17	5	
NOA	10	8.00	8.00	7.90	6.80	8.00	7.95	7.75	8.00	7.85	7.80	7.65	8.00	7.85	8.00	8	
	30	8.00	8.00	8.00	8.00	8.00	7.95	7.80	8.00	7.95	7.60	8.00	7.95	8.00	8.00	8	
	50	8.00	8.00	8.00	8.00	8.00	7.75	7.80	8.00	8.00	7.80	8.00	7.95	8.00	8.00	8	
GWO	10	5.20	4.70	4.25	5.20	3.80	3.40	4.00	3.95	2.95	3.40	4.10	3.35	3.65	4.14	4	
	30	4.15	4.45	3.85	3.15	5.05	3.40	3.00	4.00	3.60	3.10	3.90	3.15	3.90	3.72	3	
	50	3.05	4.15	3.20	2.15	2.50	2.00	2.05	4.05	2.85	3.05	4.05	3.00	4.10	3.21	3	
IGWO	10	2.80	2.75	3.20	**3.30**	3.30	2.25	**2.75**	**1.55**	3.15	1.65	**2.25**	2.35	**1.95**	2.21	2	
	30	2.30	**1.45**	1.85	2.15	2.00	**1.55**	**1.50**	2.20	2.05	**1.45**	2.00	1.70	2.00	2.00	2	
	50	2.40	3.10	2.55	3.15	5.35	3.05	2.95	**1.60**	3.85	1.95	1.60	2.10	2.15	2.59	2	
AGWO	10	6.70	5.35	5.60	3.70	6.10	6.05	3.45	5.35	5.85	5.70	4.75	5.90	5.15	5.97	6	
	30	5.85	6.35	6.25	5.75	4.75	5.75	4.90	5.10	5.10	5.35	5.05	5.90	5.70	5.86	6	
	50	5.30	5.45	5.45	5.90	5.45	5.45	4.85	4.95	5.25	5.25	5.00	5.20	5.15	5.52	6	
HSB-GWO	10	2.30	**1.05**	1.70	3.80	**1.45**	**1.65**	3.15	2.30	**1.30**	**1.45**	2.65	**1.25**	2.40	**1.66**	**1**	
	30	**1.70**	1.80	**1.45**	**1.30**	**1.00**	1.70	1.70	**1.15**	**1.80**	1.55	**1.05**	**1.35**	**1.00**	**1.17**	**1**	
	50	**1.15**	**1.05**	**2.10**	**1.00**	**1.45**	**1.00**	**1.05**	**1.60**	**1.50**	**1.15**	**1.50**	**1.00**	**1.00**	**1.14**	**1**	

**Bold font** denotes the best result.

**Table 9:** Overall rank by Friedman test in IEEE CEC 2019.

Alg.	F1	F2	F3	F4	F5	F6	F7	F8	F9	F10	Avg. Rank	Overall Rank
AO	2.80	3.70	5.00	5.00	4.35	4.95	5.00	5.35	5.70	3.85	4.67	5
AOA	2.80	7.05	7.00	6.15	6.95	7.05	5.90	6.65	5.35	3.20	6.11	6
CBOA	**2.50**	2.55	**1.30**	2.70	2.25	2.75	2.05	2.55	3.50	1.95	2.39	2
NOA	8.00	7.95	8.00	8.00	8.00	7.95	8.00	8.00	8.00	8.00	8.00	8
GWO	6.40	5.10	3.15	2.15	3.40	3.25	3.55	3.85	2.65	6.45	4.33	4
IGWO	6.60	5.90	3.45	4.00	3.45	2.75	3.55	**1.85**	2.80	5.15	3.22	3
AGWO	4.40	**1.60**	5.90	6.55	6.05	6.00	6.65	5.85	6.75	5.80	6.11	6
HSB-GWO	**2.50**	2.15	2.20	**1.45**	**1.55**	**1.30**	**1.30**	1.90	**1.25**	**1.60**	**1.17**	**1**

**Bold font** denotes the best result.

**Table 10:** Parameter setting for the cooperative path planning Task 1 for multiple UAVs.

Objective	Parameter	Symbol	Value
UAV	The number of multiple UAVs	*M*	10
	Instruction time (s)	$t_{\mathrm{com}}$	160
	Minimum and maximum airspeed (m/s)	$\left[v_{\min},v_{\max}\right]$	[102,238]
	Minimum and maximum flight altitude (km)	$\left[H_{\min},H_{\max}\right]$	[0.02,20]
	Map area (km^2^)	$N_{O}\;\times \;M_{O}$	25×25
	Minimum safe flight distance (km)	$D_{K}$	0.5
	Initial position	$S_{1}$	[1.5,1.5,15.0]
		$S_{2}$	[0.0,1.5,15.0]
		$S_{3}$	[1.5,0.0,15.0]
		$S_{4}$	[0.0,3.0,15.0]
		$S_{5}$	[3.0,0.0,15.0]
		$S_{6}$	[0.0,0.0,15.0]
		$S_{7}$	[0.0,4.5,15.0]
		$S_{8}$	[4.5,0.0,15.0]
		$S_{9}$	[1.5,3.0,15.0]
		$S_{10}$	[3.0,1.5,15.0]
	Goal position	$G_{1}$	[20.0,20.0,4.0]
		$G_{2}$	[16.0,20.0,5.0]
		$G_{3}$	[20.0,16.0,5.0]
		$G_{4}$	[18.0,20.0,4.5]
		$G_{5}$	[20.0,18.0,4.5]
		$G_{6}$	[18.0,18.0,5.0]
		$G_{7}$	[16.0,18.0,5.5]
		$G_{8}$	[18.0,16.0,5.5]
		$G_{9}$	[14.0,20.0,5.5]
		$G_{10}$	[20.0,14.0,5.5]
HSB-GWO	Weight factors for cost function Equation (7)	$\left[\omega_{1},\cdots ,\omega_{5}\right]$	[1,1,0.12,0.84,0.7]
	Proportional factors	$\left[p_{E},p_{H_{1}},p_{H_{2}}\right]$	[0.05,0.05,0.05]
	Maximum number of fitness evaluations	MaxFEs	2400
	Population size	$N_{p}$	60
	Dividing probability	$r_{E}$	0.2

**Table 11:** Threat design for the cooperative path planning Task 1 for multiple UAVs.

Objective	No.	Center Coordinates (km)	Maximum Detection Radius $D_{\max}^{R}$ (km)	Effective Detection Radius $D_{\min}^{R}$ (km)
Radar threat	1	[5.0,5.0,17.0]	2.5	1.0
	2	[15.0,7.0,10.0]	2.0	1.0
	3	[17.0,18.0,8.0]	2.0	1.0
	4	[8.0,8.0,0.0]	6.8	1.0
	5	[8.0,18.0,8.0]	3.0	1.0
Other threat	1	[4.0,11.0,12.0]	2.5	1.0
	2	[10.0,2.0,10.0]	2.0	1.0
	3	[5.0,6.0,10.0]	1.5	1.0
	4	[10.0,8.0,12.0]	1.8	1.0
	5	[11.0,13.0,13.0]	2.3	1.0
	6	[13.0,14.0,9.0]	1.8	1.0
	7	[18.0,13.0,10.0]	2.2	1.0
	8	[16.0,16.0,0.0]	4.0	1.0

**Table 12:** Parameter settings for the cooperative path planning Task 2 for multiple UAVs.

Objective	Parameter	Symbol	Value
UAV	The number of multiple UAVs	*M*	8
	Instruction time (s)	$t_{\mathrm{com}}$	6850
	Minimum and maximum airspeed (m/s)	$\left[v_{\min},v_{\max}\right]$	[102,238]
	Minimum and maximum flight altitude (km)	$\left[H_{\min},H_{\max}\right]$	[0.02,20]
	Map area (km^2^)	$N_{O}\;\times \;M_{O}$	900×900
	Minimum safe flight distance (km)	$D_{K}$	25.0
	Initial position	$S_{1}$	[0.0,0.0,10.0]
		$S_{2}$	[0.0,100.0,10.0]
		$S_{3}$	[0.0,200.0,10.0]
		$S_{4}$	[0.0,300.0,10.0]
		$S_{5}$	[0.0,100.0,15.0]
		$S_{6}$	[0.0,200.0,15.0]
		$S_{7}$	[0.0,300.0,15.0]
		$S_{8}$	[0.0,400.0,15.0]
	Goal position	$G_{1}$	[875.0,875.0,15.0]
		$G_{2}$	[800.0,875.0,15.0]
		$G_{3}$	[875.0,800.0,15.0]
		$G_{4}$	[800.0,800.0,15.0]
		$G_{5}$	[875.0,875.0,10.0]
		$G_{6}$	[800.0,875.0,10.0]
		$G_{7}$	[875.0,800.0,10.0]
		$G_{8}$	[800.0,800.0,10.0]
HSB-GWO	Weight factors for cost function Equation (7)	$\left[\omega_{1},\cdots ,\omega_{5}\right]$	[1,1,0.12,0.84,0.7]
	Proportional factors	$\left[p_{E},p_{H_{1}},p_{H_{2}}\right]$	[0.05,0.05,0.05]
	Maximum number of fitness evaluations	MaxFEs	2400
	Population size	$N_{p}$	60
	Dividing probability	$r_{E}$	0.2

**Table 13:** Threat design for the cooperative path planning Task 2 for multiple UAVs.

Objective	No.	Center Coordinates (km)	Maximum Detection Radius $D_{\max}^{R}$ (km)	Effective Detection Radius $D_{\min}^{R}$ (km)
Radar threat	1	[200.0,200.0,10.0]	20.0	1.0
	2	[600.0,500.0,10.0]	30.0	1.0
Other threat	1	[120.0,40.0,10.0]	40.0	1.0
	2	[300.0,150.0,10.0]	40.0	1.0
	3	[200.0,600.0,10.0]	40.0	1.0
	4	[380.0,450.0,10.0]	50.0	1.0
	5	[720.0,650.0,10.0]	20.0	1.0
	6	[600.0,760.0,10.0]	20.0	1.0

**Table 14:** Fitness results of 8 algorithms in 10 trials for Task 1.

	AO	AOA	CBOA	NOA	GWO	IGWO	AGWO	HSB-GWO
Tri. 1	40.85	86.80	39.67	64.17	33.22	33.64	34.67	31.38
Tri. 2	39.31	74.82	32.39	35.90	33.11	32.22	33.62	29.87
Tri. 3	40.90	88.44	33.81	41.84	32.89	33.58	34.59	31.01
Tri. 4	40.97	97.45	38.10	70.02	31.86	32.63	34.26	31.53
Tri. 5	40.76	96.81	38.70	69.87	32.24	33.16	33.76	31.09
Tri. 6	43.24	75.96	33.58	65.60	32.05	32.15	35.04	31.42
Tri. 7	41.08	65.41	36.58	74.23	31.69	33.00	34.44	30.98
Tri. 8	42.00	45.49	34.59	50.69	32.25	32.71	33.83	30.37
Tri. 9	36.63	77.03	33.57	63.72	33.55	34.47	33.94	28.90
Tri. 10	38.88	78.71	36.72	47.93	32.27	32.97	33.72	31.57
Mean	40.46	78.69	35.77	58.39	32.51	33.05	34.19	**30.81**
STD	1.82	15.44	2.52	13.25	0.63	0.71	0.48	0.86
*p*-value	1.05 $\times \;10^{-11}$	1.23 $\times \;10^{-8}$	1.41 $\times \;10^{-5}$	3.59 $\times \;10^{-6}$	8.60 $\times \;10^{-5}$	5.31 $\times \;10^{-6}$	2.68 $\times \;10^{-9}$	N/A

*Boxed font* denotes the trial with the smallest fitness of each algorithm. **Bold font** denotes the best result.

**Table 15:** Information about Task 1 of the best trial of each algorithm.

Algorithm	Best Trial	Fitness	Index	UAV Number									
				1	2	3	4	5	6	7	8	9	10
AO	Tri. 9	36.63	Distance (km)	37.39	30.71	34.82	30.25	36.50	35.10	28.36	32.00	34.65	34.83
			Arrival time (s)	366.61	129.04	341.33	296.54	185.89	344.12	278.09	313.71	339.73	341.49
			Velocity (m/s)	102.00	238.00	102.00	102.00	196.38	102.00	102.00	102.00	102.00	102.00
			Collision time	3									
AOA	Tri. 8	45.49	Distance (km)	30.40	28.57	31.04	33.92	31.64	29.10	25.39	29.70	26.40	31.74
			Arrival time (s)	295.34	277.55	301.55	329.49	307.38	282.72	246.62	288.54	256.47	308.34
			Velocity (m/s)	102.93	102.93	102.93	102.93	102.93	102.93	102.93	102.93	102.93	102.93
			Collision time	21									
CBOA	Tri. 2	32.39	Distance (km)	37.39	37.10	36.09	37.67	36.17	37.99	35.42	36.30	35.22	35.64
			Arrival time (s)	157.10	155.87	151.62	158.26	151.96	159.60	148.82	152.51	148.00	149.77
			Velocity (m/s)	238.00	238.00	238.00	238.00	238.00	238.00	238.00	238.00	238.00	238.00
			Collision time	5									
NOA	Tri. 2	35.90	Distance (km)	36.17	35.59	35.61	35.76	34.66	37.15	32.84	32.19	33.05	33.20
			Arrival time (s)	152.01	149.60	149.66	150.31	145.66	156.12	138.04	135.30	138.90	139.53
			Velocity (m/s)	237.93	237.93	237.93	237.93	237.93	237.93	237.93	237.93	237.93	237.93
			Collision time	18									
GWO	Tri. 7	31.69	Distance (km)	29.83	28.84	27.96	30.52	27.95	28.18	24.54	24.88	23.95	26.30
			Arrival time (s)	129.88	134.93	123.23	139.26	124.00	136.05	110.08	140.89	140.48	117.00
			Velocity (m/s)	229.68	213.75	226.91	219.20	225.36	207.14	222.98	176.62	170.47	224.75
			Collision time	1									
IGWO	Tri. 6	32.15	Distance (km)	30.25	31.03	29.19	28.85	28.75	29.79	25.86	26.19	24.23	25.16
			Arrival time (s)	135.99	150.00	136.28	143.83	138.52	136.83	111.21	127.35	131.69	125.74
			Velocity (m/s)	222.43	206.87	214.21	200.61	207.57	217.69	232.58	205.66	184.00	200.12
			Collision time	0									
AGWO	Tri. 2	33.62	Distance (km)	32.49	29.84	27.78	29.16	28.84	30.07	26.05	24.74	27.69	25.28
			Arrival time (s)	154.98	144.96	137.47	139.63	134.62	157.97	146.94	131.03	141.77	113.13
			Velocity (m/s)	209.65	205.84	202.10	208.85	214.21	190.38	177.28	188.82	195.34	223.45
			Collision time	7									
HSB-GWO	Tri. 9	28.90	Distance (km)	32.03	27.13	30.68	32.04	31.59	33.63	32.20	30.56	23.92	25.05
			Arrival time (s)	143.42	119.30	135.99	145.60	132.75	174.83	135.31	130.62	100.50	111.72
			Velocity (m/s)	223.31	227.38	225.63	220.03	237.93	192.34	238.00	233.94	238.00	224.19
			Collision time	0									

**Table 16:** Fitness results of 8 algorithms in 10 trials for Task 2.

	AO	AOA	CBOA	NOA	GWO	IGWO	AGWO	HSB-GWO
Tri. 1	6.12	7.35	18.96	26.05	4.96	5.47	19.66	2.80
Tri. 2	20.79	4.57	3.16	16.82	4.29	5.45	5.61	2.79
Tri. 3	17.32	15.75	19.28	9.47	4.91	5.02	9.83	2.77
Tri. 4	20.09	4.55	3.90	14.66	5.62	3.88	9.19	1.42
Tri. 5	6.13	31.86	4.95	27.28	3.51	13.26	7.74	1.40
Tri. 6	6.11	22.76	15.79	15.76	6.96	6.32	10.58	2.14
Tri. 7	10.31	4.54	3.18	14.80	3.53	6.42	7.75	1.39
Tri. 8	18.01	24.15	4.57	14.36	6.97	3.73	6.33	1.44
Tri. 9	22.21	12.25	12.99	13.95	6.32	4.20	9.22	2.81
Tri. 10	21.50	22.06	4.58	13.99	3.49	5.87	7.07	2.11
Mean	14.86	14.98	9.14	16.71	5.06	5.96	9.30	2.11
STD	6.88	9.84	6.27	5.58	1.38	2.74	3.97	0.65
*p*-value	2.16 $\times \;10^{-4}$	2.90 $\times \;10^{-3}$	7.00 $\times \;10^{-3}$	1.96 $\times \;10^{-5}$	1.29 $\times \;10^{-4}$	2.90 $\times \;10^{-3}$	1.89 $\times \;10^{-4}$	N/A

*Boxed font* denotes the trial with the smallest fitness of each algorithm.

**Table 17:** Information about Task 2 of the best trial of each algorithm.

Algorithm	Best Trial	Fitness	Index	UAV Number							
				1	2	3	4	5	6	7	8
AO	Tri. 6	6.11	Distance (km)	1307.80	1301.27	1449.27	1495.10	1279.55	1304.74	1397.60	1463.04
			Arrival time (s)	5494.96	6381.16	14,208.56	6281.92	8372.36	5482.10	7292.52	10,412.09
			Velocity (m/s)	238.00	203.92	102.00	238.00	152.83	238.00	191.65	140.51
			Collision time	8							
AOA	Tri. 7	4.54	Distance (km)	1257.47	1250.41	1186.64	1154.19	1255.02	1252.13	1188.37	1153.66
			Arrival time (s)	12,328.17	12,258.88	11,633.73	11,315.55	12,304.11	12,275.80	11,650.66	11,310.40
			Velocity (m/s)	102.00	102.00	102.00	102.00	102.00	102.00	102.00	102.00
			Collision time	6							
CBOA	Tri. 2	3.16	Distance (km)	1282.20	1245.13	1186.95	1189.22	1247.96	1253.43	1194.18	1157.35
			Arrival time (s)	5387.41	5231.62	4987.18	4996.74	5243.51	5266.50	5017.55	4862.81
			Velocity (m/s)	238.00	238.00	238.00	238.00	238.00	238.00	238.00	238.00
			Collision time	4							
NOA	Tri. 3	9.47	Distance (km)	1391.63	1254.98	1152.50	1101.74	1357.55	1234.77	1142.67	1362.81
			Arrival time (s)	12,566.71	9977.36	11,299.03	9303.92	13,309.35	12,105.55	9249.78	11,548.74
			Velocity (m/s)	110.74	125.78	102.00	118.42	102.00	102.00	123.53	118.01
			Collision time	20							
GWO	Tri. 10	3.49	Distance (km)	1406.72	1263.25	1237.32	1010.96	1416.94	1194.01	1208.92	1263.04
			Arrival time (s)	6363.44	6171.11	5793.65	4305.97	6240.14	5347.25	5988.90	6105.79
			Velocity (m/s)	221.06	204.70	213.56	234.78	227.07	223.29	201.86	206.86
			Collision time	12							
IGWO	Tri. 8	3.73	Distance (km)	1324.70	1277.09	1215.11	1103.47	1265.63	1172.50	1141.13	1075.13
			Arrival time (s)	6176.45	6046.98	5249.46	4718.92	6588.83	5294.08	5145.58	6196.69
			Velocity (m/s)	214.48	211.20	231.47	233.84	192.09	221.47	221.77	173.50
			Collision time	4							
AGWO	Tri. 2	5.61	Distance (km)	1343.72	1207.66	1134.77	1153.67	1327.50	1237.80	1133.24	1101.12
			Arrival time (s)	6948.94	6371.08	5115.83	6955.72	7823.53	6708.25	5672.00	5547.25
			Velocity (m/s)	193.37	189.55	221.81	165.86	169.68	184.52	199.79	198.50
			Collision time	21							
HSB-GWO	Tri. 7	1.39	Distance (km)	1252.06	1177.78	1079.71	969.49	1260.54	1202.49	1100.11	1039.80
			Arrival time (s)	5290.13	4970.26	5479.77	4131.14	6859.77	6848.08	6372.45	4464.42
			Velocity (m/s)	236.68	236.97	197.04	234.68	183.76	175.60	172.64	232.91
			Collision time	4							

**Table 18:** Fitness results of three ablation algorithms and HSB-GWO in 10 trials using Task 1.

	A-1	A-2	A-3	HSB-GWO
Tri. 1	35.02	36.17	33.31	31.38
Tri. 2	35.07	35.49	35.01	29.87
Tri. 3	34.30	35.50	36.15	31.01
Tri. 4	38.58	35.32	35.69	31.53
Tri. 5	64.41	34.65	35.41	31.09
Tri. 6	61.53	35.45	36.30	31.42
Tri. 7	34.91	33.60	36.15	30.98
Tri. 8	35.14	36.41	34.85	30.37
Tri. 9	34.20	34.67	35.40	28.90
Tri. 10	35.50	34.93	36.44	31.57
Mean	40.86	35.22	35.47	**30.81**
STD	11.73	0.81	0.93	0.86
*p*-value	1.46 $\times \;10^{-2}$	6.54 $\times \;10^{-10}$	8.86 $\times \;10^{-10}$	N/A

*Boxed font* denotes the trial with the smallest fitness of each algorithm. **Bold font** denotes the best result.

## Data Availability

Dataset available on request from the authors.
